# Structural basis for an unprecedented enzymatic alkylation in cylindrocyclophane biosynthesis

**DOI:** 10.7554/eLife.75761

**Published:** 2022-02-25

**Authors:** Nathaniel R Braffman, Terry B Ruskoski, Katherine M Davis, Nathaniel R Glasser, Cassidy Johnson, C Denise Okafor, Amie K Boal, Emily P Balskus

**Affiliations:** 1 Department of Chemistry and Chemical Biology, Harvard University Cambridge United States; 2 Department of Biochemistry and Molecular Biology, The Pennsylvania State University University Park United States; 3 Department of Chemistry, The Pennsylvania State University University Park United States; 4 Howard Hughes Medical Institute, Harvard University Cambridge United States; Johns Hopkins University School of Medicine United States; Harvard Medical School United States

**Keywords:** X-ray crystallography, natural product biosynthesis, aromatic alkylation, alkyl halide, cyanobacteria, Cylindrospermum licheniforme, *E. coli*

## Abstract

The cyanobacterial enzyme CylK assembles the cylindrocyclophane natural products by performing two unusual alkylation reactions, forming new carbon–carbon bonds between aromatic rings and secondary alkyl halide substrates. This transformation is unprecedented in biology, and the structure and mechanism of CylK are unknown. Here, we report X-ray crystal structures of CylK, revealing a distinctive fusion of a Ca^2+^-binding domain and a β-propeller fold. We use a mutagenic screening approach to locate CylK’s active site at its domain interface, identifying two residues, Arg105 and Tyr473, that are required for catalysis. Anomalous diffraction datasets collected with bound bromide ions, a product analog, suggest that these residues interact with the alkyl halide electrophile. Additional mutagenesis and molecular dynamics simulations implicate Asp440 in activating the nucleophilic aromatic ring. Bioinformatic analysis of CylK homologs from other cyanobacteria establishes that they conserve these key catalytic amino acids, but they are likely associated with divergent reactivity and altered secondary metabolism. By gaining a molecular understanding of this unusual biosynthetic transformation, this work fills a gap in our understanding of how alkyl halides are activated and used by enzymes as biosynthetic intermediates, informing enzyme engineering, catalyst design, and natural product discovery.

## Introduction

Chemists utilize alkyl halides (molecules with sp^3^ C–X bonds, X = F, Cl, Br, or I) as key synthetic reagents because of their accessibility and favorable reactivity (Rudolph and Lautens, 2009); however, examples of their use as intermediates in biological systems are rare (Adak and Moore, 2021). A few characterized natural product biosynthetic pathways generate transiently halogenated intermediates that facilitate downstream chemical reactions in a strategy known as ‘cryptic halogenation’ (Vaillancourt et al., 2005). This biosynthetic logic requires two partner enzymes: a halogenase, which introduces the alkyl halide substituent, and a second enzyme that utilizes the halogenated intermediate as a substrate, leveraging its increased reactivity and releasing the corresponding halide anion as a side product. Multiple classes of halogenases have been structurally and mechanistically characterized (Neumann et al., 2008), but the cognate halide-utilizing enzymes in pathways that employ cryptic halogenation remain underexplored. In particular, the structures and biochemical mechanisms for enzymatically engaging and activating alkyl halide substrates as intermediates in natural product biosynthesis are unknown.

**Figure 1.**
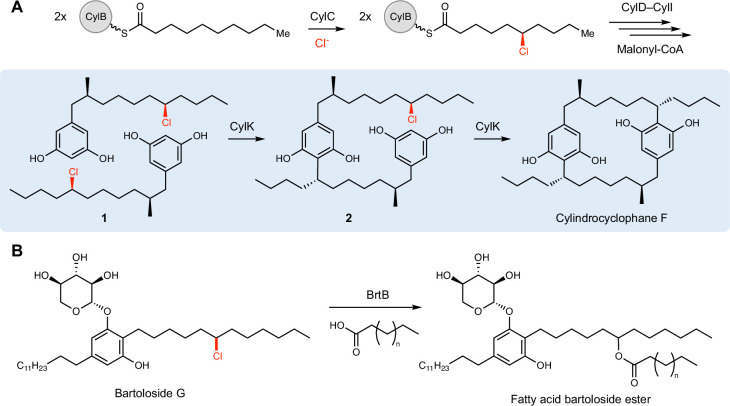
CylK and related enzymes use alkyl chloride substrates as biosynthetic intermediates. (**A**) The halogenase CylC generates an alkyl chloride substrate for CylK, which catalyzes two stepwise Friedel–Crafts alkylations to construct a paracyclophane macrocycle in cylindrocyclophane biosynthesis. This involves an intermolecular reaction between two resorcinol-containing alkyl chloride substrates (**1**) to generate intermediate (**2**), followed by an intramolecular alkylation to afford cylindrocyclophane F. (**B**) The related enzyme BrtB (30% amino acid identity, 46% similarity) catalyzes an analogous but chemically distinct C–O bond-forming event between chlorinated bartolosides and fatty acid nucleophiles.

Interrogating cylindrocyclophane biosynthesis by the cyanobacterium *Cylindrospermum licheniforme* ATCC 29412 presents an exciting opportunity to study alkyl halide utilization in biological systems. In this organism, the putative diiron carboxylate halogenase CylC generates an alkyl chloride intermediate, which is further elaborated to produce resorcinol-containing alkyl chloride **1**. Two molecules of **1** are then dimerized by an alkyl chloride-utilizing enzyme, CylK, via the formation of two new carbon–carbon (C–C) bonds to construct a paracyclophane ring system (Figure 1A; Nakamura et al., 2017). CylK is annotated as a fusion of a Ca^2+^-binding domain and a β-propeller fold, but the roles of these predicted protein domains in catalysis are unknown. Initial biochemical studies revealed that this transformation involves two stereoselective alkylation events that occur in a stepwise fashion, with inversion of configuration at the alkyl chloride stereocenter (Nakamura et al., 2017). CylK is the only enzyme known to catalyze aromatic ring alkylation with an alkyl halide electrophile, a reaction that mirrors a classical nonenzymatic reaction known as the Friedel–Crafts alkylation, which is an important transformation in organic synthesis. The traditional nonenzymatic Friedel–Crafts reaction suffers from a lack of stereo- and regiocontrol, unwanted overalkylation events, and the generation of carbocationic intermediates that can undergo unproductive rearrangements (Friedel and Crafts, 1877; Roberts and Khalaf, 1984).

In contrast, the CylK-catalyzed Friedel–Crafts alkylation overcomes these limitations, likely by tightly controlling reactivity within the enzyme active site. This enzymatic transformation holds promise for biocatalytic applications, and we have recently demonstrated CylK’s ability to accept multiple discrete resorcinol nucleophiles and alkyl halide electrophiles with varying substitution and electronic character (Schultz et al., 2019). Moreover, uncharacterized enzymes with homology to CylK are found in numerous cyanobacteria, one of which (BrtB) was recently revealed to catalyze carbon–oxygen (C–O) bond formation between the carboxylate groups of fatty acids and bartoloside A, an alkyl chloride-containing natural product (Figure 1B; Reis et al., 2020). This suggests that the mechanism by which CylK binds and activates alkyl chlorides might be shared with this and other homologs that use diverse nucleophilic substrates.

Despite the intriguing reactivity and potential applications of CylK and related alkyl halide activating enzymes, our knowledge of its structure and mechanism is limited. In this work, we present a crystal structure of CylK, identify critical active site residues, provide experimental and bioinformatic support for their roles in catalysis, and propose a mechanism by which alkyl chloride substrates are activated for stereospecific nucleophilic substitution. In determining how CylK performs this Friedel–Crafts alkylation, we have enhanced our fundamental understanding of how enzymes engage alkyl halide substrates. Our structural information and mechanistic model will guide future enzyme engineering efforts, inform the design of nonenzymatic catalysts, and enable genome mining to uncover new natural products constructed by related biosynthetic strategies.

## Results

### CylK is a distinctive fusion of two protein domains

**Figure 2.**
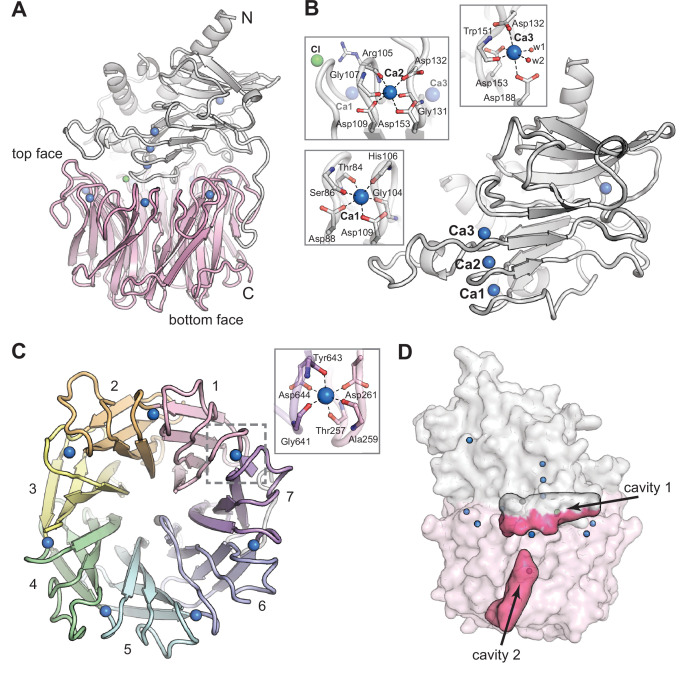
The CylK X-ray crystal structure reveals a distinct arrangement of two protein domains. (**A**) An overall view of the structure of CylK. In this image and throughout, calcium ions are shown as blue spheres, magnesium ions as dark gray spheres, and chloride ions as green spheres. The N-terminal domain is depicted as a light gray ribbon diagram, and the C-terminal domain is shown as a pink ribbon diagram. (**B**) The N-terminal domain contains a right-handed parallel β-roll stabilized by three Ca^2+^ ions. The structure is capped by a three-strand antiparallel β-sheet and buttressed by additional helical secondary structures. Insets show the Ca^2+^ ion coordination environment within the β-roll. (**C**) A top-down view of the seven-bladed β-propeller C-terminal domain. The propeller blades are numbered from the N-terminal end of the domain and colored by blade. The inset shows a representative view of the Ca^2+^ coordination environment. A similar binding site exists at each blade junction. (**D**) Cavity mapping analysis (Ho and Gruswitz, 2008) of CylK reveals two cavities large enough to accommodate the alkyl resorcinol substrates.

CylK crystallized in the *C*222_1_ space group with a single monomer in the asymmetric unit (Table 1). Suitable molecular replacement (MR) models for phasing could not be identified. Phase information was obtained by partial substitution of native Ca^2+^-binding sites in CylK with Tb^3+^, followed by collection of X-ray diffraction datasets at the Tb X-ray absorption peak energy. The structure of CylK was solved to 1.68 Å resolution via a combined MR and single-wavelength anomalous diffraction (SAD) approach, with the MR search model generated from a poly-alanine seven-bladed β-propeller. The structure reveals an unprecedented fusion of two protein folds, an N-terminal Ca^2+^-binding domain and a C-terminal β-propeller domain (Figure 2A).

**Figure 2—figure supplement 1.**
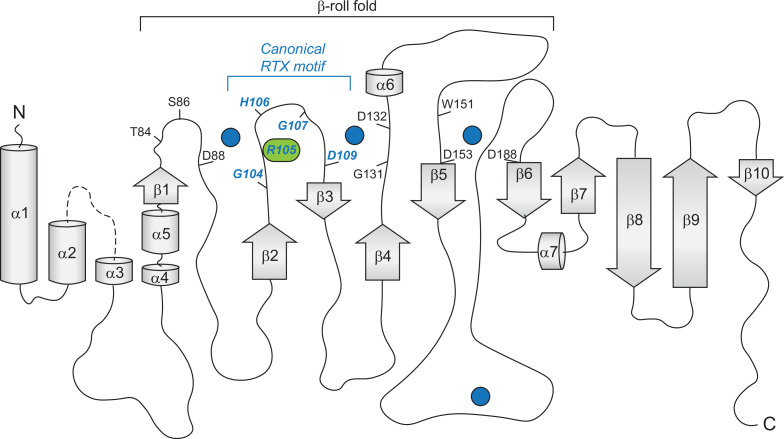
Topology diagram of the N-terminal CylK β-roll domain, residues 7–251. Ca^2+^ ions are shown as blue circles, residues implicated in catalysis are highlighted in green ovals, Ca^2+^-coordinating residues are shown in black, and Ca^2+^-coordinating residues that follow canonical repeat-in-toxin (RTX) motifs are shown in blue italics.

**Figure 2—figure supplement 2.**
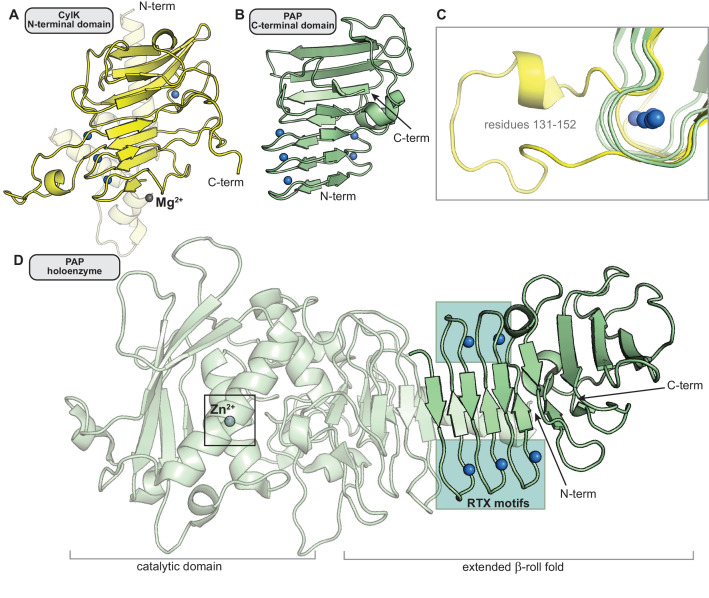
Comparison of the N-terminal CylK β-roll domain to similar C-terminal repeat-in-toxin (RTX) domains in other enzymes. (**A**) The N-terminal domain of CylK (residues 7–251). (**B**) The C-terminal region of RTX-motif enzyme psychrophilic alkaline metalloprotease (PAP) that structurally aligns with the β-roll of CylK (residues 326–461) (PDB ID 1OMJ). (**C**) Some CylK Ca^2+^-coordinating loops (yellow, residues 131–152) extend beyond the tight RTX-motif turn (green, PAP residues 330–335, 348–353, and 366–371). (**D**) The Ca^2+^-binding β-roll motif in PAP is located far away from the active site of the enzyme (box) and is likely not involved in catalysis (Ravaud et al., 2003). In all panels, Ca^2+^ ions are represented as blue spheres. Mg^2+^ (CylK) and Zn^2+^ (PAP) ions are shown as gray spheres.

**Figure 2—figure supplement 3.**
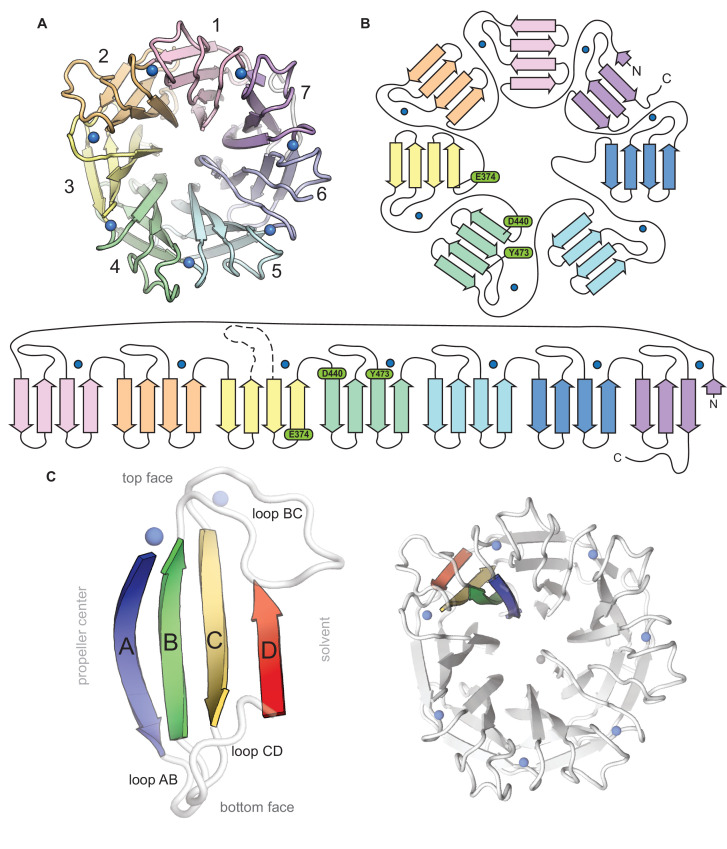
Topological and structural features of the C-terminal CylK β-propeller domain. (**A**) Top-down view of the C-terminal β-propeller domain (residues 252–662). (**B**) Topology diagrams of the CylK β-propeller domain. Blades are colored as in (**A**), Ca^2+^ ions shown as blue circles. Residues implicated in catalysis are shown in green. (**C**) Example of a propeller blade (left) and the orientation of the blade within the β-propeller fold (right). β-strands within each blade are lettered A–D, with A corresponding to the innermost strand, closest to the central tunnel. Loops are labeled to indicate the strands they connect. For example, loop BC connects blade B and C within the blade while loop DA connects strand D of one blade to strand A of the next blade.

**Figure 2—figure supplement 4.**
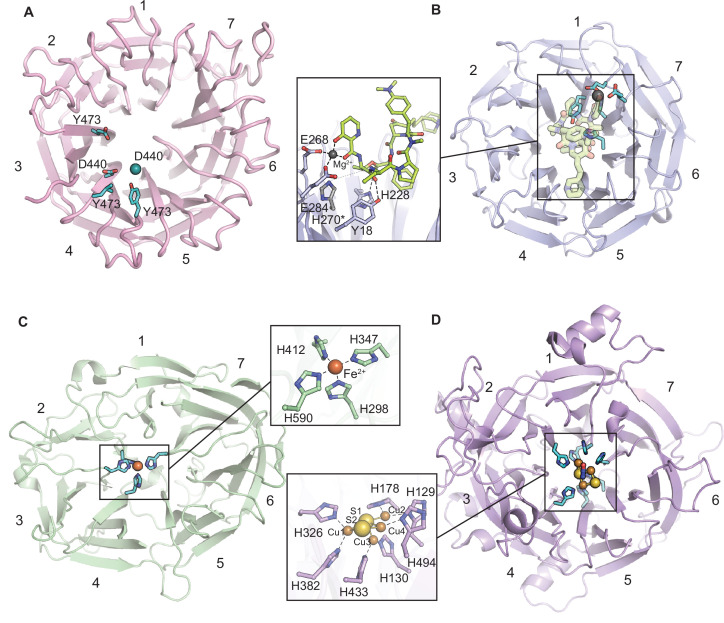
Comparison of the proposed CylK active site location to those of other characterized seven-bladed β-propeller enzymes. Panels show a top-down ribbon diagram of β-propeller domains. Residues implicated in catalysis are shown as cyan sticks. (**A**) The C-terminal domain of CylK. Br^-^ ions are shown as teal spheres, and Ca^2+^ ions are shown as transparent blue spheres. (**B**) Virginiamycin B lyase (VrgB) complexed with the substrate analog, quinupristin (lime green sticks) (PDB ID 2Z2P). Mg^2+^ ions are shown as gray spheres. *H270 modeled as in PDB ID 2Z2O. (**C**) Carotenoid cleavage dioxygenase (CCD) (PDB ID 3NPE). Fe^2+^ ions are shown as orange spheres. (**D**) Nitrous oxide reductase (NOR) complexed with N_2_O (blue and red sticks) (PDB ID 3SBR) and the active site Cu-S cluster. Cu ions are shown as orange spheres, and sulfur atoms are shown as yellow spheres.

**Figure 2—figure supplement 5.**
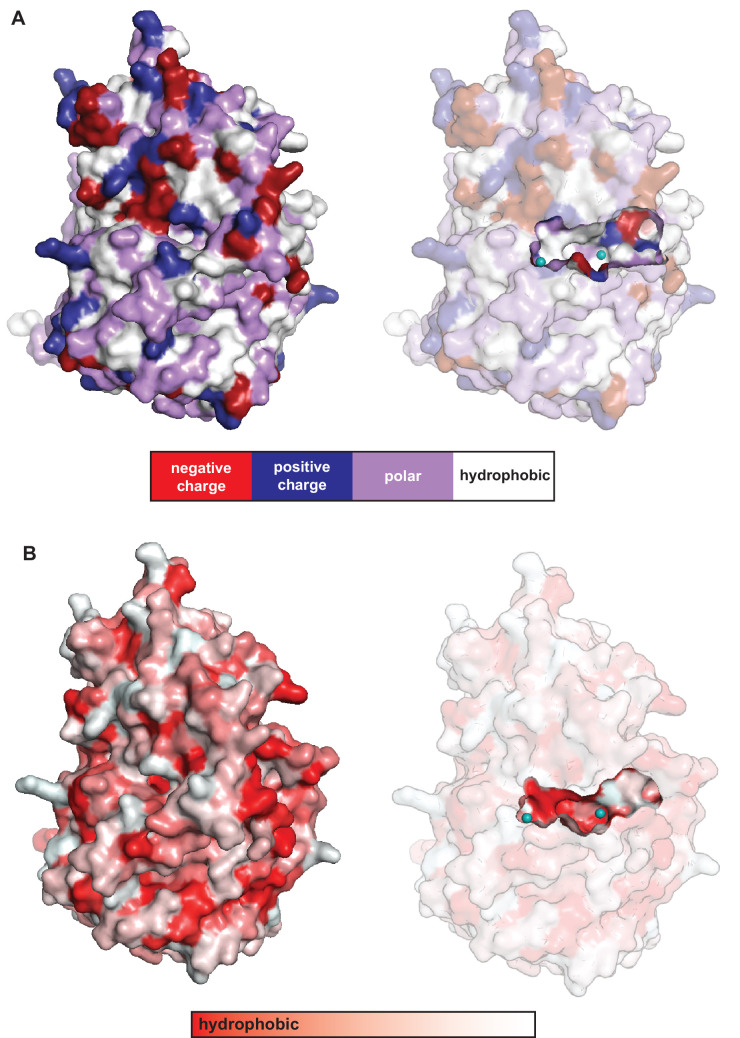
Solvent accessibility and properties of the cavity located in between the C-terminal β-propeller domain and the N-terminal domain in CylK. The walls of this cavity contain (**A**) charged (red/blue) and polar (purple) residues and (**B**) hydrophobic patches (red), ideal for interaction with the amphipathic resorcinol.

**Figure 2—figure supplement 6.**
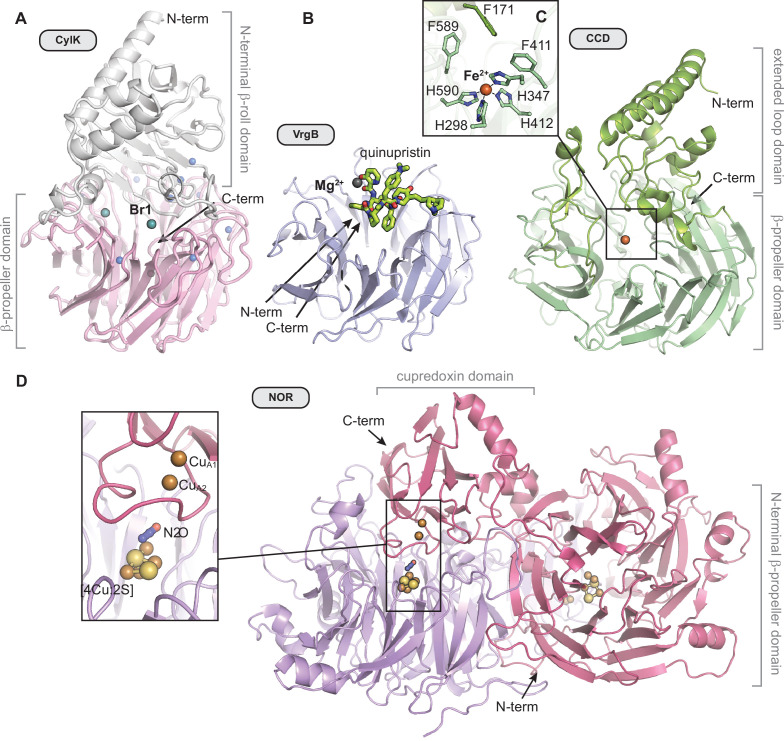
A comparison of the overall structures of CylK and selected seven-bladed β-propeller enzymes. (**A**) CylK is a fusion of a Ca^2+^-binding β-roll fold and a β-propeller with the active site located at the domain interface. (**B**) Virginiamycin B lyase (VrgB) is a single-domain enzyme in which the active site is located on the top face of the propeller. Quinupristin substrate analog shown as green sticks (PDB ID 2Z2P). (**C**) The extended loops of carotenoid cleavage dioxygenase (CCD) form a helical domain on the top face of the propeller, oriented similarly to the N-terminal domain of CylK. The active site, delineated by the location of the iron cofactor (orange sphere), is located at the interface of these two domains (PDB ID 3NPE). (**D**) Dimeric nitrous oxide reductase (NOR) contains an active site at the interface between a C-terminal cupredoxin electron transfer domain (Cu_A_) contributed by the other monomer and the β-propeller catalytic domain (inset) (PDB ID 3SBR). Unless otherwise noted, ions are shown as spheres and colored as in Figure 2—figure supplement 2.

**Table 1.** Data collection and refinement statistics for Tb-soaked *C. licheniforme* CylK structures.

	Tb anomalous	Native (final model)
**Data collection**		
Space group	*C* 2 2 2_1_	*C* 2 2 2_1_
Wavelength (Å)	1.6314	0.97872
Cell dimensions		
*a*, *b*, *c* (Å)	94.265, 139.286, 100.381	93.667, 138.613, 99.438
α, β, γ (°)	90.0, 90.0, 90.0	90.0, 90.0, 90.0
Resolution (Å)	50–2.38 (2.42–2.38)	50.0–1.68 (1.71–1.68)
*R*_merge_	0.218 (0.670)	0.101 (1.326)
*R*_pim_	0.041 (0.192)	0.026 (0.346)
*I* / σ*I*	25.2 (3.8)	29.3 (2.15)
CC_1/2_	1.010 (0.906)	0.999 (0.780)
Completeness (%)	99.5 (99.9)	99.8 (99.3)
Redundancy	29.5 (11.4)	14.8 (14.3)
**Refinement**		
Resolution (Å)		42.41–1.68
No. reflections		68,476
*R*_work_/*R*_free_		0.1556/0.1894
No. atoms		5,292
Protein		4,903
Ligand/ion		50
Water		339
*B*-factors (Å^2^)		
Protein		18.20
Ligand/ion		28.54
Water		25.82
RMS deviations		
Bond lengths (Å)		0.016
Bond angles (°)		1.921
Molprobity clashscore		2.29 (99th percentile)
Rotamer outliers (%)		0.78
Ramachandran favored (%)		96.33
Ramachandran allowed (%)		3.51
Ramachandran outlier ^*^(%)		0.16
PDB accession code		7RON

* Values in parentheses are for highest-resolution shell.

The N-terminal domain of CylK contains a short helical component that packs against a β-roll core (Figure 2B, Figure 2—figure supplement 1). The core fold is structurally similar to repeat-in-toxin (RTX) motifs, found in diverse bacterial extracellular proteins (Linhartová et al., 2010). A search of the PDB for structural relatives of the CylK N-terminal domain reveals similarity to the C-terminal RTX domains of secreted toxins (Motlova et al., 2020), such as the adenylate cyclase toxin of *Bordetella pertussis*, and bacterial surface-layer proteins (von Kügelgen et al., 2020), such as the RsaA protein of *Caulobacter crescentus* (Table 2). In these systems, the RTX domain facilitates Ca^2+^-dependent folding or assembly in the calcium-rich environment outside the cell but not in the calcium-depleted cytosol. The CylK N-terminal domain also resembles RTX domains fused to catalytic domains of extracellular hydrolases and epimerases from Gram-negative bacteria (Figure 2—figure supplement 2; Tanaka et al., 2007; Buchinger et al., 2014; Meier et al., 2007). In these systems, the RTX unit is attached to the C-terminus of the catalytic domain to facilitate extracellular secretion and Ca^2+^-dependent folding. The RTX component of these characterized proteins is functionally modular, with no obvious direct connection to the active site, although in some systems that target large biopolymer substrates, the RTX domain may help anchor the substrate via electrostatic interactions (Buchinger et al., 2014).

**Table 2.** Selected structural homologs of the N-terminal domain of CylK obtained by a structural comparison to other proteins in the PDB (Gáspári, 2020).

Rank	PDB ID	Functional description	Z-score	RMSD	No. residues aligned	Total residues
1	1OMJ-A	Psychrophilic alkaline metalloprotease (PAP); serralysin family	11.2	6.8	135	456
2	2Z8Z-A	MIS38 lipase	11.1	8.5	116	616
3	6SUS-A	RTX domain of blocks IV and V of adenylate cyclase toxin	11.0	3.1	120	258
4	2QUA-A	LipA, extracellular lipase from *Serratia marcescens*	10.6	2.3	110	615
5	2ML1-A	AlgE6R1, Mannuronan C5-epimerase	10.6	3.0	122	153

The N-terminal domain of CylK differs from typical RTX motifs in several important ways. Canonical RTX motifs have an extended compact oval structure shaped by tight turns between subsequent β-strands that are stabilized by stacked metal-binding motifs on both sides of the core fold (Linhartová et al., 2010). The metal-binding sites are formed by a repeating GXXGXD motif in the tight turn. Vertically stacked pairs of these repeats yield hexacoordinate Ca^2+^-binding sites with four carbonyl ligands and two carboxylates provided by the conserved Asp side chains. CylK adopts a more asymmetric version of this fold and contains just a single copy of the consensus Ca^2+^-binding motif. The modified β-roll structure in CylK contains three vertically aligned Ca^2+^ ions on one side of the core fold. The other side lacks the metal-binding sites found in other RTX proteins. Additionally, while the first two turns of the β-roll in CylK form tight junctions near the Ca^2+^ ions, all of the subsequent turns contain long extensions (Figure 2—figure supplement 1), some of which facilitate integration with the C-terminal domain. Interestingly, the section of the β-roll motif that conserves the Ca^2+^ sites packs closely into the center of the C-terminal domain. Although the CylK N-terminal domain is topologically distinct from other β-roll motifs, CylK shares with these systems a functional requirement for Ca^2+^ (Nakamura et al., 2017).

The C-terminal domain of CylK adopts a seven-bladed β-propeller fold (Figure 2C, Figure 2—figure supplement 3), a widespread structural motif found in both eukaryotic and prokaryotic protein structures (Chen et al., 2011). In eukaryotes, this flat, cylindrically shaped fold facilitates protein–protein interactions (Schapira et al., 2017) via the same interface (top face) that interacts with the N-terminal domain in CylK. In prokaryotes and some eukaryotes (plants), β-propeller folds can be used in catalysis (Chen et al., 2011). Comparison of the CylK β-propeller domain to other examples of this fold in bacterial enzymes reveals several structural differences unique to CylK (Figure 2—figure supplement 4). In all β-propellers, adjacent four-stranded ‘blades’ are connected back-to-front by loops on the top face of the domain. The top face of the fold also consists of loops that link the internal β-strands of each propeller blade. In CylK, these internal loops are unusually long and folded back over the outside of the central propeller fold. Also, the loops that connect subsequent blades contain unique blade-bridging Ca^2+^-binding sites, suggesting a shared role for this divalent metal in structural stabilization of both domains of CylK.

CylK is structurally similar to a class of single-domain bacterial β-propeller enzymes implicated in streptogramin antibiotic resistance that includes virginiamycin B lyase (Vgb) (Table 3; Korczynska et al., 2007; Lipka et al., 2008). These enzymes share with CylK an ability to interface with macrocyclic substrates or products, but they perform a distinct chemical transformation. While CylK forms two C–C bonds to generate a cyclic product, the streptogramin resistance proteins linearize cyclic peptide substrates by cleaving a C–O bond. X-ray crystal structures of an inactive variant of Vgb bound to a substrate analog provide insight into the location of the active site and mechanism of macrocycle opening (Korczynska et al., 2007). In Vgb, the substrate analog and an Mg^2+^ ion necessary for catalysis bind near conserved polar side chains in blades 6 and 7 of the propeller fold (Figure 2—figure supplement 4B). In CylK, these sites are substituted for hydrophobic side chains, suggesting that, despite the similarities to streptogramin lyases, the substrates of CylK likely bind in a different location and are transformed by a distinct mechanism.

**Table 3.** Selected structural homologs of the C-terminal domain of CylK obtained by a structural comparison to other proteins in the PDB (Gáspári, 2020).

Rank	PDB ID	Functional description	Z-score	RMSD	No. residues aligned	Total residues
1	1SQ9-A	Antiviral protein Ski8	31.2	2.4	286	378
2	4H5J-B	Guanine nucleotide-exchange factor Sec12	31.1	2.9	300	347
3	5TF2-A	Prolactin regulatory element-binding protein	31.0	2.7	291	338
4	4J0X-B	Ribosomal RNA-processing protein 9	30.9	2.4	290	366
5	2Z2O-C	Virginiamycin B lyase	30.5	2.4	286	299

### The CylK active site is located at the domain interface

**Figure 3.**
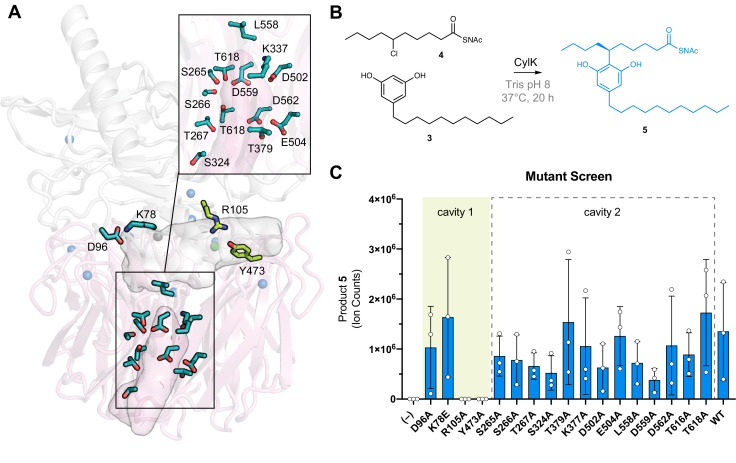
The active site of CylK is located at the domain interface. (**A**) Selected amino acids in CylK near cavities 1 and 2 subjected to mutant scanning are shown in stick format. Side chains shown in yellow near cavity 1 correspond to sites found to be essential for activity. (**B**) Non-native substrate pair used in CylK activity assays and product of the alkylation reaction (SNAc = *N*-acetylcysteamine). (**C**) Screen of mutant activity to locate the CylK active site. Product formation was measured by liquid chromatography-mass spectrometry (LC-MS), error bars represent the standard deviation from the mean of three biological replicates.

To identify CylK’s active site, we focused on two solvent-accessible cavities (Figure 2D). Cavity 1 is lined by the top face of the β-propeller domain and the N-terminal domain, forming a solvent-accessible tunnel that is ~18 Å deep and 15 Å wide at its largest point. The walls of this cavity contain both charged/polar residues and hydrophobic patches, ideal for interaction with the amphipathic resorcinol substrates (Figure 2—figure supplement 5). We also interrogated a second central channel located exclusively within the C-terminal domain and opening to the opposite (bottom face) side of the propeller motif (cavity 2). To identify the active site, we individually mutagenized 17 polar residues spanning the two cavities (Figure 3A) that are conserved in putative CylK homologs from cyanobacteria known to produce structurally related natural products (Figure 3—figure supplement 1, Table 4; Preisitsch et al., 2015; May et al., 2017; Preisitsch et al., 2016; Leão et al., 2015). To rapidly test variant proteins for activity, we designed a plate-based lysate activity assay using non-native substrate pair **3 + 4** (Figure 3B), which was previously demonstrated to be accepted by CylK (Schultz et al., 2019). All variants associated with the central/bottom channel of the C-terminal β-propeller retained full alkylation activity (Figure 3C). Strikingly, substitution of two residues located at the domain interface between the top face of the β-propeller and the N-terminal domain, Arg105 and Tyr473, completely abolished all activity on substrate pair **3 + 4** (Figure 3C). Notably, although located in close proximity, Arg105 is supplied by the N-terminal domain, while Tyr473 is from the C-terminal β-propeller, suggesting that both domains play an essential role in catalysis. These data preliminarily supported the location of the active site as the cleft formed between the two domains of CylK (cavity 1).

**Figure 3—figure supplement 1.**
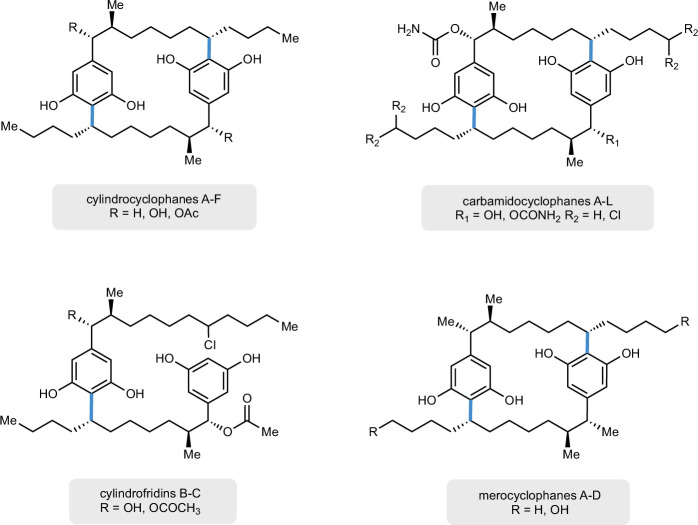
Structurally related, cylindrocyclophane-like natural products produced by cyanobacteria with related biosynthetic gene clusters and CylK homologs, namely, merocyclophanes (*Nostoc* sp. UIC 10110, MerH), cylindrofridins (*Cylindrospermum stagnale* PCC 7417, CylK), and carbamidocyclophanes (*Nostoc* sp. CAVN2, CabK). The bonds highlighted in blue are hypothesized to be constructed by their respective CylK.

**Table 4.** Conservation of CylK residues selected for mutagenesis experiments.

Position	Residue(CylK, *C. licheniforme*)	Mutant	Residue (CabK)	Residue(CylK, *C. stagnale*)	Residue(MerH)	Residue(BrtB)
78	Lys	Glu	*Lys*	*Lys*	*Lys*	*Lys*
96	Asp	Ala	*Asp*	*Asp*	*Asp*	*Asp*
105	Arg	Ala, Lys	*Arg*	*Arg*	*Arg*	*Arg*
473	Tyr	Ala,	*Tyr*	*Tyr*	*Tyr*	*Tyr*
265	Ser	Ala	*Ser*	*Ser*	Ala	Ala
266	Ser	Ala	*Ser*	*Ser*	*Ser*	Thr
267	Thr	Ala	*Thr*	*Thr*	*Thr*	Ala
324	Ser	Ala	*Ser*	*Ser*	*Ser*	*Ser*
377	Lys	Ala	Arg	*Lys*	Arg	Gly
379	Thr	Ala	*Thr*	*Thr*	*Thr*	Ala
502	Asp	Ala	*Asp*	*Asp*	*Asp*	*Asp*
504	Glu	Ala	*Glu*	*Glu*	*Glu*	Ala
558	Leu	Ala	*Leu*	*Leu*	*Leu*	Met
559	Asp	Ala	*Asp*	*Asp*	*Asp*	Gly
562	Asp	Ala	His	*Asp*	Glu	Val
616	Thr	Ala	*Thr*	*Thr*	*Thr*	Gly
618	Thr	Ala	*Thr*	*Thr*	Ser	Ser
374	Glu	Ala	*Glu*	*Glu*	*Glu*	Arg
438	Leu	Ala	*Leu*	*Leu*	*Leu*	*Leu*
440	Asp	Ala, Asn	*Asp*	*Asp*	*Asp*	Glu
499	Phe	Ala	*Phe*	*Phe*	*Phe*	Tyr
318	Ser	Ala	*Ser*	*Ser*	Thr	Ile
334	Asn	Ala	*Asn*	*Asn*	*Asn*	Trp

**Figure 4.**
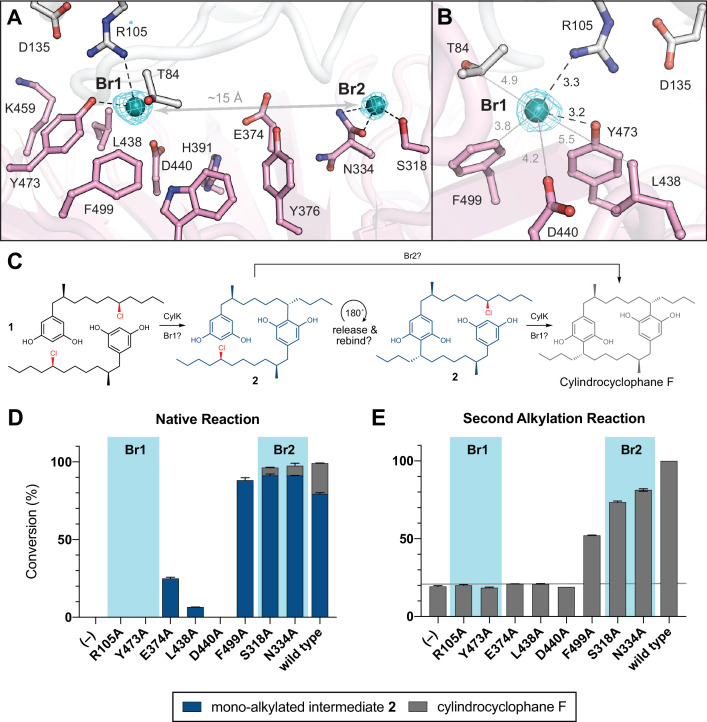
NaBr soak and mutagenesis of bromide-binding residues suggest a single site for catalysis. (**A**) A soak of NaBr into CylK crystals reveals two strong peaks in the anomalous difference electron density map (cyan mesh, contoured at 5.0 σ) within cavity 1. Bromide ion 1 (Br1) and 2 (Br2) are shown as teal spheres, and selected amino acids in the vicinity of each site are shown in stick format. The C-terminal domain is colored white, and the N-terminal domain is colored pink. (**B**) Alternate view of Br1 with anomalous difference electron density map, polar contacts shown as black dashed lines, and other distances shown as gray lines. All distances measured in angstroms. (**C**) Proposed alkylation scheme with potential roles for two bromide-binding sites in paracyclophane formation, or invoking release and rebinding of monoalkylated intermediate **2** for alkylation at a single bromide site. (**D**) End point activity at 1 hr of select mutants performing the native reaction with substrate **1**, highlighting the residues associated with Br1 or Br2. Product formation was quantified by high-performance liquid chromatography (HPLC), error bars represent the standard deviation from the mean of two biological replicates. (**E**) End point activity at 22 hr of select mutants performing the second alkylation reaction with intermediate **2**. Figure 4—source data 1.Western blot of Strep-tagged CylK mutants (from left to right): R105A, R105K, S318A, N334A, E374A, L438A; and serial dilutions of purified wild-type CylK: 2.0, 0.5, and 0.1 µM. Figure 4—source data 2.Western blot of Strep-tagged CylK mutants (from left to right): D440A, D440N, Y473A, Y473F, F499A, wild-type; and serial dilutions of purified wild-type CylK: 2.0, 0.5, and 0.1 µM.

In parallel, we hypothesized that the alkyl chloride moiety may be an important substrate-binding determinant based on our inability to obtain a full occupancy complex of resorcinol **3** in CylK crystals. Although **3** was required for CylK crystallization, we could not model the alkyl resorcinol substrate in the resulting structure. To visualize locations within CylK capable of binding an alkyl halide or free halide, we soaked CylK crystals with NaBr solutions. The Br^–^ ions are surrogates for the native Cl^–^ side product and are generated in reactions with alkyl bromide electrophiles, which CylK was previously shown to accept with similar efficiency to alkyl chlorides (Schultz et al., 2019). Bromine X-ray absorption energies can be easily accessed at a conventional synchrotron X-ray source for anomalous diffraction experiments, allowing us to pinpoint the location of the halide in the structure. Initial refinements identified two strong positive peaks located ~15 Å apart in *F*_o_−*F*_c_ electron density maps of the interdomain cavity (cavity 1). Anomalous diffraction datasets collected at the absorption edge of bromine confirmed assignment of these peaks as Br^–^ ions (Figure 4A, Table 5). Anomalous peaks were not found in the central channel of the β-propeller domain (cavity 2).

**Figure 4—figure supplement 1.**
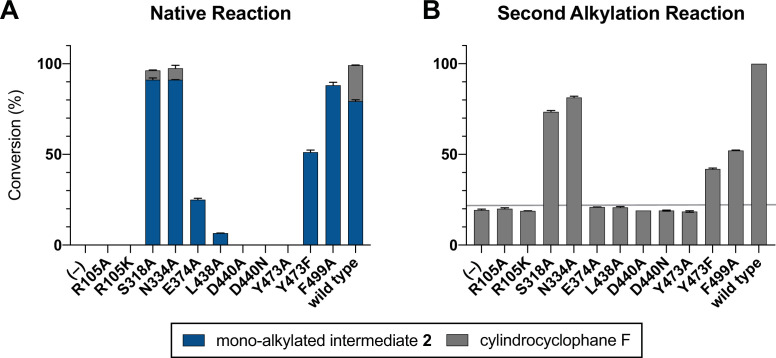
Mutagenesis of active site cleft. (**A**) End point activity at 1 hr of select mutants performing the native reaction with substrate **1**. Product formation was quantified by high-performance liquid chromatography (HPLC), error bars represent the standard deviation from the mean of two biological replicates. (**B**) End point activity at 22 hr of select mutants performing the second alkylation reaction with intermediate **2**.

**Figure 4—figure supplement 2.**
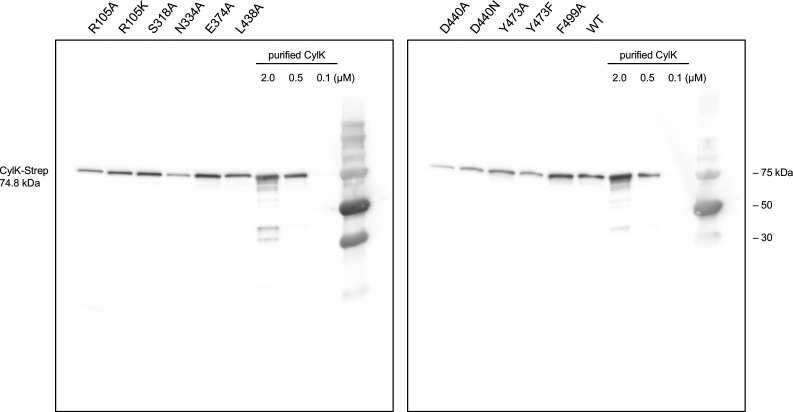
Western blotting of Strep-tagged CylK mutants from soluble lysate with anti-Strep-horseradish peroxidase (HRP, IBA) in order to ensure mutant enzyme expression and solubility. Serial dilution of purified CylK-Strep included to visualize protein concentration range.

**Table 5.** Data collection and refinement statistics for Br-soaked *C. licheniforme* CylK structures.

	Native	Br anomalous
**Data collection**		
Space group	*C* 2 2 2_1_	*C* 2 2 2_1_
Wavelength (Å)	0.97872	0.9184
Cell dimensions		
*a*, *b*, *c* (Å)	93.470, 138.656, 99.511	93.557, 138.726, 99.706
α, β, γ (°)	90.0, 90.0, 90.0	90.0, 90.0, 90.0
Resolution (Å)	50.0–1.52 (1.55–1.52)	50–1.54 (1.57–1.54)
*R*_merge_	0.076 (0.703)	0.725 (4.755)
*R*_pim_	0.029 (0.295)	0.149 (1.140)
*I* / σ*I*	23.3 (2.14)	24.0 (2.06)
CC_1/2_	0.990 (0.843)	0.978 (0.124)
Completeness (%)	99.7 (98.9)	100.0 (100.0)
Redundancy	7.0 (5.5)	24.2 (16.9)
		
**Refinement**		
Resolution (Å)	46.78–1.52	
No. reflections	89,122	
*R*_work_ / *R*_free_	0.1693/0.1978	
No. atoms	5437	
Protein	4989	
Ligand/ion	45	
Water	403	
*B*-factors (Å^2^)		
Protein	16.68	
Ligand/ion	23.03	
Water	23.77	
RMS deviations		
Bond lengths (Å)	0.013	
Bond angles (°)	1.823	
Molprobity clashscore	1.64 (99th percentile)	
Rotamer outliers (%)	0.76	
Ramachandran favored (%)	96.41	
Ramachandran allowed (%)	3.59	
Ramachandran outlier (%)	0.00	
PDB accession code	7ROO	

*Values in parentheses are for highest-resolution shell.

The bromide peak of highest intensity (~33 σ) in the anomalous difference electron density map, Br1, is located just inside the opening to the interdomain cavity (Figure 4B). Br1 is modeled at nearly full (85%) occupancy and appears to make hydrogen-bonding contacts with the side chains of Arg105 from the N-terminal domain and Tyr473 from the C-terminal domain, both residues identified in our mutagenesis scan. Notably, the apo CylK model also contained a positive *F*_o_−*F*_c_ electron density peak at the Br1 site. Given the presence of chloride salts in the protein storage buffer, we modeled this site as a chloride ion in the apo model (Figure 2B). Br1 resides within 8 Å of the calcium-binding sites in the N-terminal domain, and the backbone carbonyl of Arg105 coordinates the central Ca^2+^ ion (Ca2) in the β-roll motif of the N-terminal domain. This observation further supports an active role for both domains in the CylK-catalyzed reaction. Br1 is located within 5–6 Å of other side chains that form a portion of the solvent accessible channel nearest to the protein surface (Figure 4B), including a number of polar residues that could be implicated in activation of the resorcinol nucleophile and/or the alkyl chloride electrophile. Two residues, Asp440 and Leu438, undergo a rotamer change upon bromide binding. A second bromide peak, Br2, modeled at 48% occupancy, is located deep within the interdomain cavity. This ion makes contact with Ser318, Asn334, and Trp320, all contributed by the C-terminal domain. While residues surrounding both bromides are generally conserved in closely related CylK homologs, those associated with Br2 are not conserved in the C–O bond-forming family member BrtB (Table 4). This observation suggests that while Br2 residues might be relevant for paracyclophane-forming enzymes, they may not be necessary for all family members.

### Key catalytic residues are located at Br1 within the active site cleft

Having discovered that the CylK active site cleft has two potential alkyl halide-binding/-activating regions, we wondered if the two discrete native alkylation reactions catalyzed by CylK occurred at one or both bromide-binding sites. If only a single bromide site is catalytically active, we speculate that the monoalkylated intermediate **2** is likely released, rotates 180°, and must rebind the active site for the second alkylation event to occur (Figure 4C). To test this proposal, we individually mutagenized several residues closely associated with or appearing to directly bind either bromide, and examined their ability to perform the first and second alkylation reactions with native substrates **1** and **2**. Considering that the first alkylation is effectively faster than the second (Nakamura et al., 2017), likely due to substrate and/or product inhibition, we monitored the first alkylation by interrogating the 1 hr time point of the native reaction, when wild-type enzyme has fully transformed both equivalents of **1** to intermediate **2** (>99% conversion). Notably, substitution of the residues that comprise the Br1-binding site (Arg105, Tyr473) abolished CylK activity toward **1** at 1 hr, while mutations that disrupt Br2 (Ser318, Asn334) had no apparent effect (Figure 4D), retaining wild-type levels of activity. Variants targeting three additional residues located between the two bromide sites (Glu374, Leu438, Asp440) had very low or no activity and stood out as potential candidates for substrate binding or nucleophile activation due to their side chain functionalities and vicinity to Br1 on the cleft surface. Mutating Phe499 to Ala did not significantly reduce conversion.

We then examined the second alkylation reaction using a mixture of intermediate **2** containing a small amount of cylindrocyclophane F (~18%) as the substrate. Consistent with the results obtained for the first alkylation reaction, variants that disrupted Br1 did not have appreciable activity on intermediate **2**, while substitution of residues comprising Br2 maintained near wild-type activity (Figure 4E). Mutating Glu374, Leu438, and Asp440 also resulted in no significant activity toward **2** after 22 hr, while the Phe499 to Ala mutant retained appreciable activity as before. Of note, mutating Arg105 to Lys, and Asp440 to Asn resulted in no activity for either alkylation, suggesting that the size, hydrogen-bonding capacity, and/or pK_a_ of these residues might be important for catalysis (Figure 4—figure supplement 1). However, mutating Tyr473 to Phe only moderately reduced activity and might suggest that the aromatic nature and/or size of this residue is critical for alkylation. Based on these results, we propose that Br1 is the site of alkyl chloride binding and both aromatic ring alkylation events. Additional work is required to determine if the residues that comprise Br2 assist in substrate binding, but it is clear from these data that they are not essential for catalysis. Furthermore, the site of catalysis at Br1 is located near the outermost portion of the solvent-exposed interdomain cavity; this is in agreement with previous work that demonstrated CylK’s ability to accept non-native substrates with rigid substitutions on the resorcinol ring nucleophile (Schultz et al., 2019), which would necessitate a flexible or open active site.

### Molecular dynamics simulations suggest mode of substrate binding and activation

**Figure 5.**
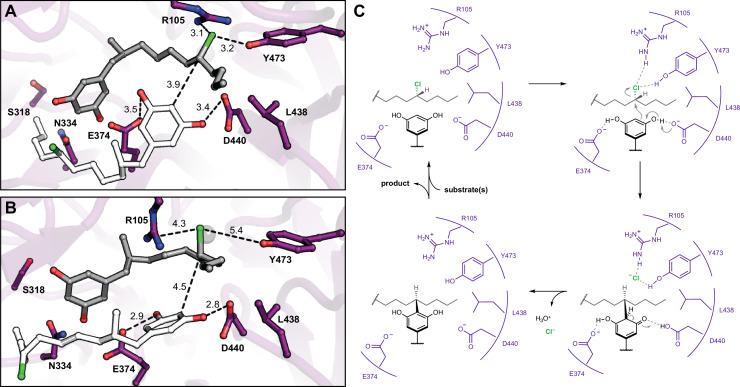
Molecular dynamics simulations reveal roles for key catalytic residues and enable a mechanistic proposal. (**A**) An energy-minimized docking model of CylK in complex with two chlorinated alkylresorcinol molecules in cavity 1 before and (**B**) after unrestrained molecular dynamics simulation. This analysis shows that both substrates can be accommodated while maintaining contact with one another and essential catalytic residues. Substrate molecules and selected amino acid side chains shown in stick format. (**C**) Proposed mechanism for a single cycle of the CylK-catalyzed Friedel–Crafts alkylation with key residues illustrated.

Having identified the site of alkylation and potential key residues, we sought to determine the specific mode of substrate binding and assess the relative contributions of specific side chains toward nucleophile (resorcinol) versus electrophile (alkyl chloride) activation. We began by computationally docking two equivalents of native substrate **1** into the active site of CylK (Figure 5A). The initial complexes were generated manually using the Br1 site to anchor the electrophilic alkyl chloride substituent of one equivalent of **1**. Previous DFT calculations performed on a model alkyl resorcinol substrate suggested that interaction between a resorcinol phenol substituent and a carboxylate hydrogen bond acceptor would enhance nucleophilicity of the aromatic ring (Schultz et al., 2019). Accordingly, potential interactions with key carboxylate residues Asp440 and Glu374 guided the placement of the nucleophilic resorcinol of the other equivalent of substrate **1**. Additionally, we oriented the nucleophilic and electrophilic carbon centers at an appropriate distance (<4 Å) and arrangement to accommodate the known stereochemical inversion of the substitution reaction. Two docked initial complexes were used, and both were run as restrained and unrestrained simulations. Restrained simulations included 1 kcal/mol·Å^2^ restraints on all protein atoms to allow substrates to search the active site. All simulations consistently demonstrated that the active site cleft can accommodate two equivalents of substrate **1**, and furthermore, Asp440 and Glu374 maintained H-bonding contacts with the resorcinol nucleophile (Figure 5B). For the majority of the simulation time, the chloride-binding pocket formed by Arg105 and Tyr473 (Br1) maintained contact with the alkyl chloride electrophile (Figure 5—figure supplement 1, Figure 5—figure supplement 2). We conclude that Asp440 and Glu374 are likely resorcinol nucleophile-interacting residues, while Arg105 and Tyr473 bind and activate the alkyl chloride electrophile with hydrogen-bonding interactions. Furthermore, the source of CylK’s exquisite regio- and stereoselectivity can be explained by the orientation in which the resorcinol nucleophile is held in close proximity to the backside of the carbon–chlorine (C–Cl) bond. Based on these results, we can propose a mechanism of alkylation that is distinct from chemical and enzymatic precedent (Figure 5C). Simulations of intermediate **2**, positioned for the second alkylation reaction, maintained similar alkyl chloride electrophile interactions; however, the resorcinol nucleophile did not remain at an appropriate distance or orientation for the reaction (Figure 5—figure supplement 3). More work is required to accurately model the second alkylation step; however, it is clear from our mutagenic work that the same residues are essential for both alkylation events.

**Figure 5—figure supplement 1.**
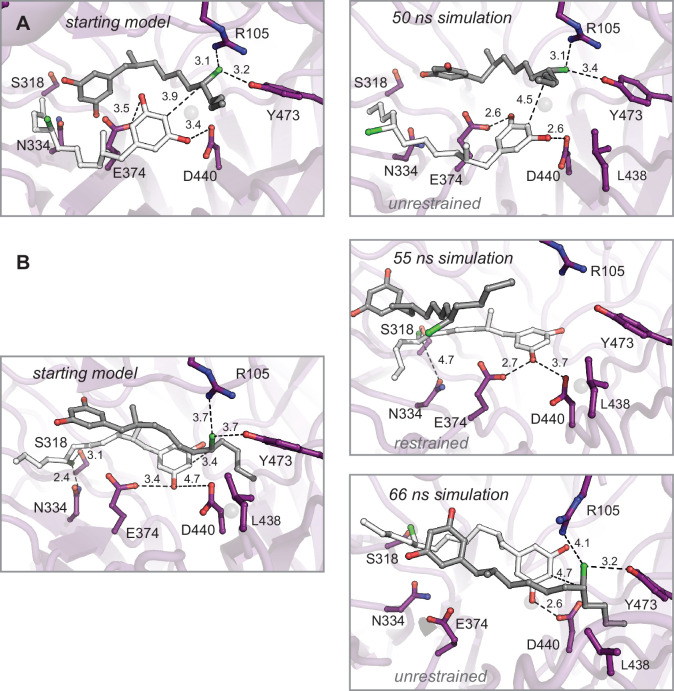
Molecular dynamics simulations of enzyme–substrate complexes manually docked into interdomain cavity support active site assignment in this region of CylK. (**A**) An energy-minimized starting model before (left) and after restrained molecular dynamics simulation (right). The complex was generated using Br1 to place one equivalent of substrate **1** (gray). This complex also maximizes the interaction between the resorcinol of a second substrate molecule (white) and amino acids D440 and E374 in the propeller domain. Results from an unrestrained simulation are shown in the main text (Figure 4D and E). (**B**) Restrained (top right) and unrestrained (bottom right) simulations resulting from a starting model (left) that prioritized Br2 site interactions. Select residues shown as dark purple sticks, distances shown as dashed lines and measured in angstroms.

**Figure 5—figure supplement 2.**
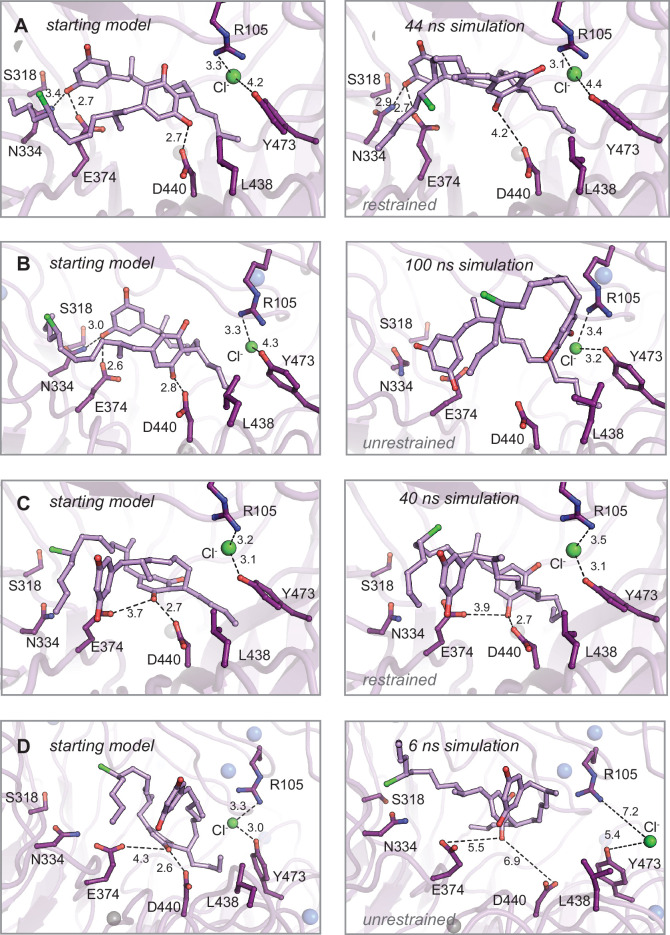
Molecular dynamics simulations of intermediate product **2** complexes manually docked into interdomain cavity reveal a halide-binding pocket involving R105 and Y473. Energy-minimized starting models before (left) and after simulation (right). (**A**) Restrained and (**B**) unrestrained simulations of the intermediate product complex. (**C**) Restrained and (**D**) unrestrained simulations of the intermediate product complex in an alternate starting conformation. Select residues shown as dark purple sticks, product intermediate shown as light purple sticks, and distances shown as dashed lines and measured in angstroms.

**Figure 5—figure supplement 3.**
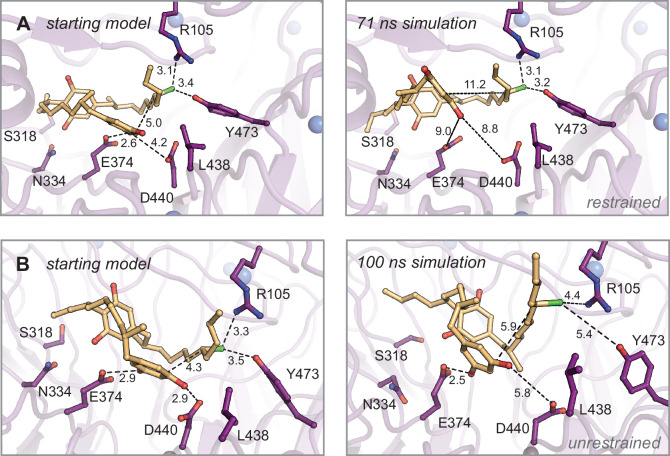
Molecular dynamics simulations of monoalkylated intermediate **2** positioned for the second alkylation reaction. Energy-minimized starting models before (left) and after simulation (right). (**A**) Restrained and (**B**) unrestrained simulations of (**A**). Select residues shown as dark purple sticks, monoalkylated intermediate **2** shown as orange sticks, and distances shown as dashed lines and measured in angstroms.

**Figure 5—figure supplement 4.**
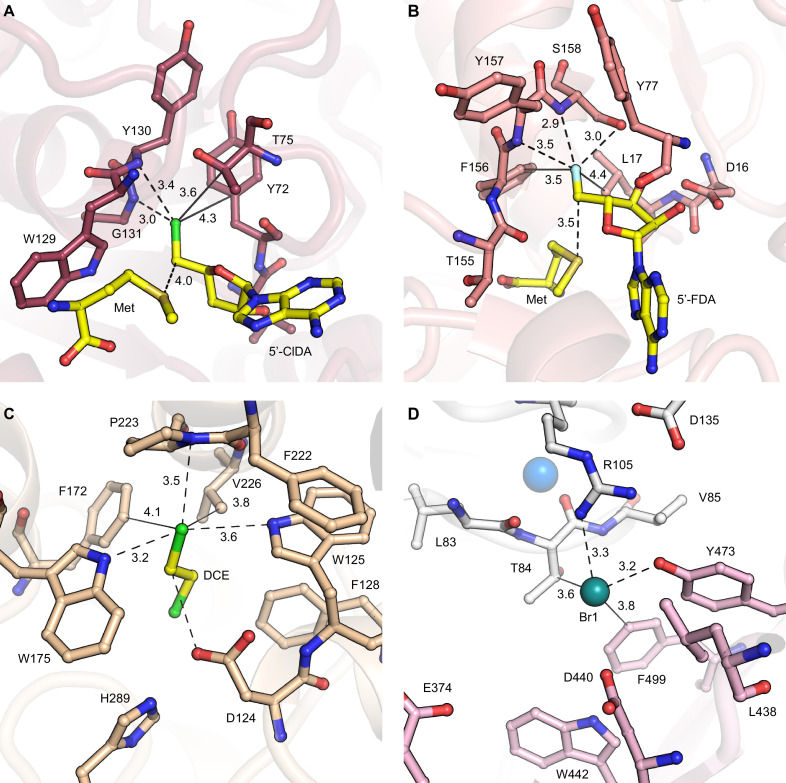
Comparison of bound alkyl halides and their respective enzyme active sites. (**A**) 5′-Chloro-5′-deoxyadenosine (5’-ClDA) and methionine (Met) products bound in the active site of wild-type SalL SAM-dependent chlorinase (PDB ID 2Q6I). The alkyl chloride forms apparent polar interactions (dashed lines) with the backbone amides of Gly 131 and Tyr 130; and nonpolar interactions (solid lines) with Tyr 72 and Thr 75. (**B**) 5′-fluoro-5′-deoxyadenosine (5’-FDA) and Met products bound in the active site of wild-type FlA SAM-dependent fluorinase (PDB ID 1RQR). The alkyl fluoride forms apparent polar interactions with the backbone amides of Ser 158 and Tyr 157, and the side chain of Ser 158; and nonpolar interactions with Phe 156 and Leu 17. (**C**) 1,2-Dichloroethane (DCE) substrate bound in the active side of wild-type haloalkane dehalogenase (PDB ID 2DHC). The alkyl chloride forms apparent polar interactions with Trp 125, 175, and the backbone amide of Pro 223; and nonpolar interactions with Phe 172 and Val 226. Asp 124 is the nucleophilic residue that displaces chloride. (**D**) Bromide ion product analog bound in the active site of CylK. The Br^-^ ion forms polar interactions with Arg 105 and Tyr 473; and nonpolar interactions with Thr 84 and Phe 499. Select amino acid residues are represented in stick format, bromide ions are shown as teal spheres, calcium ions are shown as blue spheres, alkyl chloride in green, and alkyl fluoride in light aqua.

### Bioinformatic analysis supports proposed residues implicated in alkyl chloride activation

**Figure 6.**
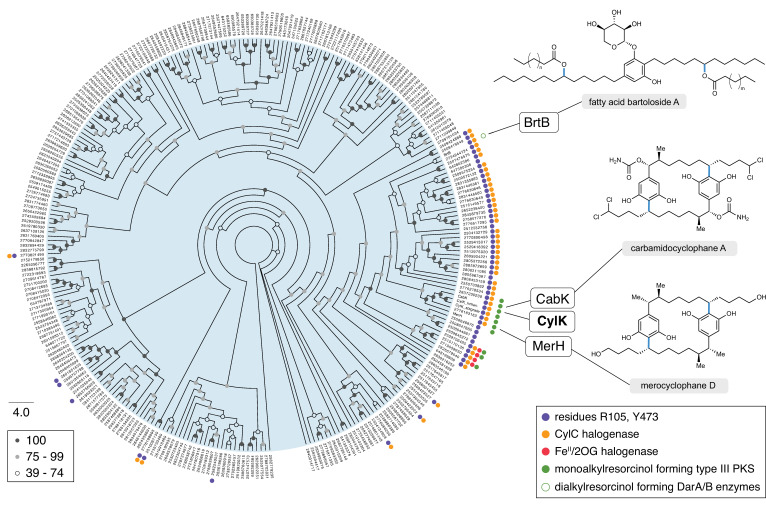
CylK homologs with partner CylC halogenases contain the proposed alkyl chloride-activating residues. Maximum-likelihood phylogenetic tree of protein sequences homologous to CylK and BrtB that contain both N- and C-terminal domains (>24% amino acid identity), highlighting the key conserved catalytic residues, clustered halogenases, and associated monoalkylresorcinol-forming enzymes. Dialkylresorcinol-forming enzymes (DarA/B-like) were not found except as expected with BrtB and the bartoloside-producing cluster, indicated as an unfilled green circle. Bootstrap values are shaded based on the legend. Natural products associated with select CylK homologs are displayed; the bonds highlighted in blue are constructed by their respective CylK.

We next sought to apply the functional insights gained from our structural analysis of CylK to improve our understanding of less closely related family members. Previously, bioinformatically identifying CylK-like enzymes was challenging because proteins encoding β-propeller motifs are common in publicly available protein databases, making it unclear which hits were true family members. With the knowledge that both protein domains of CylK are necessary for catalysis, we could now accurately locate CylK-like enzymes encoded in sequenced genomes. From a BLAST search, we identified over 700 proteins with >24% amino acid identity to CylK or BrtB (Supplementary file 1). That group was pared down to 286 unique enzyme sequences containing both N- and C-terminal domains found in CylK, and their relationship was assessed by constructing a maximum-likelihood phylogenetic tree (Figure 6).

**Figure 6—figure supplement 1.**
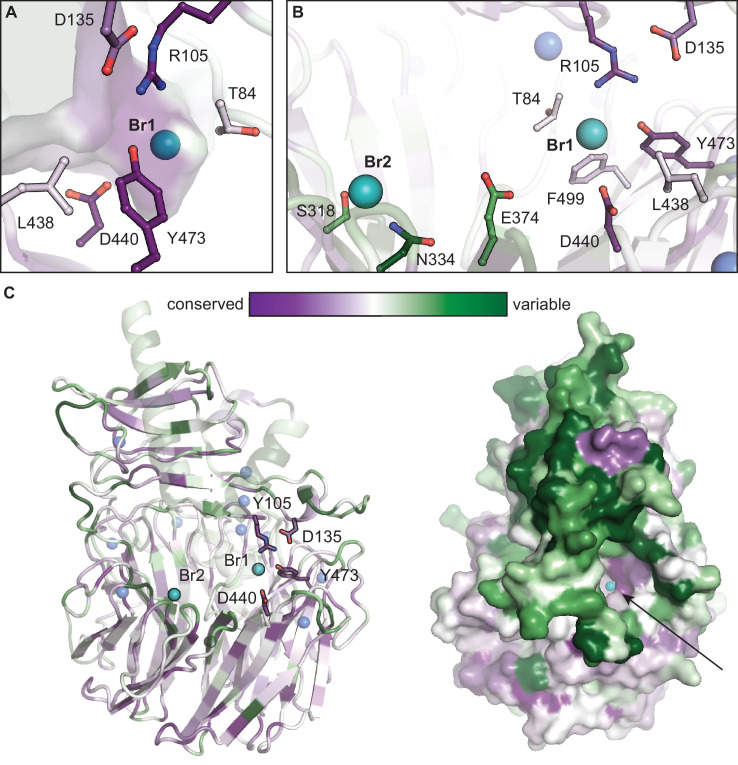
Conservation mapping analysis of functionally annotated CylK homologs. Representations of sequence conservation generated in ConSurf (Ashkenazy et al., 2016) using a subset of cyanobacterial CylK homologs that have R105 and Y473 (48 unique sequences). Of those homologous enzymes, 37 contain a partner CylC halogenase in the genome of the encoding organism (~80% are co-localized with CylK). (**A**) The halide-binding pocket in CylK homologs with R105 and Y473. (**B**) Residues surrounding Br1 are generally conserved in this subset, while Leu 438, Glu 374, and other active site residues are not highly conserved, potentially suggesting a diversity of substrates used by these candidate enzymes. Residues surrounding Br2 are variable. (**C**) Ribbon and surface diagrams of overall sequence conservation in this subset of homologs. Selected amino acid residues are represented in stick format, bromide ions are shown as teal spheres, and calcium ions are shown as blue spheres. Arrow denotes the opening of the interdomain cavity proposed to contain the active site of CylK.

We further analyzed these data by looking for the presence or absence of a partner CylC halogenase encoded in the same genome. A subset of CylK homologs that are mostly clustered together on the phylogenetic tree are found in organisms that also have a putative CylC halogenase. Of these CylK and CylC enzyme pairs, ~80% are co-localized in their respective genomes, hinting that they might work together within a biosynthetic pathway (Supplementary file 2). We found very few other types of halogenases co-localized with CylK homologs; those identified were of the iron(II) 2-(oxo)-glutarate (Fe^II^/2OG)-dependent enzyme family but were also clustered with CylC halogenases. We examined each putative CylK sequence for the presence of the proposed alkyl chloride-activating residues Arg105 and Tyr473. Intriguingly, nearly all of the candidate CylK enzymes containing both key residues were from organisms that also encode a CylC halogenase (42 of 53 organisms), suggesting that they are likely using halogenated substrates and that Arg105 and Tyr473 are indeed important for alkyl chloride activation. This subset of CylK homologs was aligned and visualized (Figure 6—figure supplement 1), which revealed that, while the resorcinol nucleophile-activating residue Asp440 was also highly conserved, Leu438, Glu374, and other active site residues are not highly prevalent within this subset. This demonstrates that the correlation between Arg105, Tyr473, and the presence of a CylC halogenase is not simply due to the overall similarity of this subset of enzymes.

Inspired by the biosynthetic logic of the *cyl* pathway, we also looked for co-localized monoalkylresorcinol (MAR)-forming enzymes encoded near putative CylK homologs in order to ascertain if resorcinols are likely to serve as their native nucleophilic substrates. To our knowledge, biosyntheses of MARs only involve type III PKS enzymes such as CylI (Martins et al., 2019). Surprisingly, we found only 10 CylI homologs clustered with CylK homologs, representing just 19% of the candidate enzymes that have Arg105 and Tyr473 residues and are expected to have alkyl chloride-activating activity. This observation suggests that a broader diversity of nucleophilic substrates might be used by these uncharacterized alkyl chloride-activating enzymes. Of note, dialkylresorcinol-forming enzymes such as those involved in bartoloside biosynthesis were not found in CylK-encoding gene clusters beyond the bartoloside gene cluster. The observation that the majority of CylK homologs do not have the proposed alkyl chloride-activating residues, partner halogenases, nor resorcinol forming enzymes, indicates that this enzyme family may catalyze other reactions not involving alkyl halides or resorcinols. This information will enable future studies by prioritizing organisms that might produce more distantly related natural products and/or CylK-like enzymes with alternate substrate scopes.

## Discussion

Our results provide a structural basis for understanding the unusual enzymatic Friedel–Crafts alkylation catalyzed by CylK in cylindrocyclophane biosynthesis. This structural model, together with supporting biochemical experiments and bioinformatic analyses, has provided fundamental insight into this intriguing reaction that represents the only known example of aromatic ring alkylation with an alkyl halide electrophile. The molecular knowledge we have gained may be applied to access and engineer novel biocatalysts, design chemocatalysts, and discover new natural products that may be candidate therapeutics and/or play important physiological roles in cyanobacteria. We note that a recently published paper also described the structure of CylK and reached similar conclusions (Wang et al., 2022).

We identified the active site of CylK at the interface of its N- and C-terminal domains. Together with previously characterized enzymes, our results show how structurally related β-propeller enzymes have divergently adapted this fold for catalytic function. In two metalloenzymes, carotenoid cleavage dioxygenase (CCD) (Messing et al., 2010) and nitrous oxide reductase (NOR) (Pomowski et al., 2011), an active site metallocofactor is lodged on the top face of the propeller and coordinated by His side chains from inner β-strands of the fold (Figure 2—figure supplement 4). Like CylK, both of these enzymes have a capping motif on the top face of the propeller. CCD contains more extensive internal loops than CylK, and these adopt structured helix-loop motifs that bury the top face of the propeller. NOR is a di-domain dimeric enzyme that employs an electron transfer domain from an adjacent monomer to shield the catalytic metallocofactor in the β-propeller motif. In both, these domain arrangements create a favorable environment for substrate binding and catalysis, as we observe in CylK (Figure 2—figure supplement 6).

Both domains of CylK have several bound calcium ions, a row of which is near the proposed alkyl chloride-binding site. However, the closest Ca^2+^ ion is approximately 8 Å from this pocket. Although we have previously demonstrated a functional requirement for calcium (Nakamura et al., 2017), our structural work suggests that a direct role in catalysis is unlikely. Removing calcium might cause a significant conformational change that precludes access to or alters the architecture of the active site as the treatment of CylK with ethylenediaminetetraacetic acid (EDTA) was observed to cause a shift in oligomeric state from monomer to a dimer. This might be indicative of larger structural changes within each unit of CylK that are incompatible with catalysis. It is unknown if calcium plays a regulatory role in vivo, although it is a component of the cyanobacterial BG-11 growth medium.

We propose a plausible mechanism for CylK catalysis that invokes positioning the reactive partners in close proximity for a concerted S_N_2-like reaction, consistent with the known inversion of configuration at the alkyl chloride stereocenter upon substitution (Figure 5C). In particular, we suggest that the side chain carboxylates of Asp440 and Glu374 hydrogen bond with the two phenol substituents of the resorcinol nucleophile. By deprotonating one phenol, the essential residue Asp440 could enhance the nucleophilicity of the reactive carbon. Analogous deprotonation of a resorcinol has been invoked as a critical step in an enzymatic Friedel–Crafts acylation reaction with acetyl arene electrophiles, lending support to our proposal (Pavkov‐Keller et al., 2018). Simultaneously, we hypothesize that Arg105, Tyr473, Phe499, and the hydrophobic portion of Thr84 form an alkyl chloride-binding pocket as observed in the bromide-soaked CylK structure. Hydrogen-bonding interactions between the polar residues and the alkyl chloride could weaken the C–Cl bond, reducing the overall activation energy barrier for substitution. Interestingly, mutating Tyr473 to Phe only slightly reduces alkylation activity, which suggests that the alkyl chloride Tyr473 interaction might resemble an anion–pi interaction (Schottel et al., 2008; Garau et al., 2003). While there is precedent for alkyl halide–arene interactions (Parisini et al., 2011), they are less thoroughly studied than anion–pi interactions. Alternatively, if Tyr473 provides a key hydrogen bond, the Phe mutant may partially compensate by forming an alkyl halide–pi interaction. In either case, this alkyl chloride-binding pocket is reminiscent of the active sites of the *S*-adenosylmethionine (SAM)-dependent chlorinase (Eustáquio et al., 2008) and fluorinase (Dong et al., 2004) enzymes. In these structures, similar ensembles of polar and aliphatic residues bind and stabilize a halide anion as it reacts with SAM in a S_N_2 substitution reaction that resembles the reverse of the transformation catalyzed by CylK (Figure 5—figure supplement 4A and B). Interestingly, our proposed CylK alkyl chloride-binding mode is distinct from that of the well-studied haloalkane dehalogenase enzyme, which primarily uses the indole N–H bonds of adjacent Trp residues to activate a primary alkyl chloride for substitution by the side chain carboxylate of an active site Asp via hydrogen bonding (Figure 5—figure supplement 4C; Verschueren et al., 1993). We speculate that the differences between these two enzymes arise from their distinct evolutionary histories as well as the decreased hydrophobicity and increased reactivity of a primary alkyl chloride substrate. Following alkylation, the chloride anion product diffuses out of the active site, and the aromaticity of the resorcinol is restored upon proton transfer to water, perhaps via His391. Finally, intermediate **2** is released from the active site, must reorient 180°, and rebind for the second intramolecular alkylation to occur in the same fashion. This reorientation likely requires significant energy to disrupt protein–substrate interactions and might suggest active site conformational changes occur to accommodate intermediate **2** in the catalytically competent orientation; however, this warrants further investigation. Although we cannot rule out a radical mechanism, the architecture of the active site and lack of radical initiation sources suggests that one-electron processes are unlikely.

We have emphasized the distinction between the proposed resorcinol nucleophile-activating residues and those that likely activate the alkyl chloride electrophile in order to compare CylK with the divergent C–O bond-forming family member, BrtB (30% amino acid identity, 46% similarity). We hypothesize that both enzymes utilize a similar strategy to enhance the reactivity of their alkyl chloride substrates, although it remains to be determined if BrtB is similarly stereoinvertive. In agreement with this proposal, BrtB contains Arg105 and Tyr473 equivalents that may play a role in alkyl chloride activation. In place of the nucleophile-activating Asp440, BrtB contains a functionally similar Glu, and notably, Glu374 is substituted with a positively charged Arg. These two residues might interact with carboxylate nucleophiles, although the precise roles of these residues in BrtB’s distinct reactivity remain to be determined. Interestingly, a carboxylate nucleophile would be predominantly deprotonated at physiological pH, suggesting that nucleophile activation might be unnecessary in this enzyme. Regardless, the divergent reactivity of CylK and BrtB hints that other relatives might perform unique chemistry and may even be amenable to protein engineering efforts to alter their substrate scopes and reactivity.

The reaction catalyzed by CylK represents a unique approach for enzymatic C–C bond formation. Other biological strategies to form analogous arene–alkyl C–C bonds involve radical intermediates that must be precisely held and oriented in their enzyme active sites in order to prevent side reactions. Examples include the radical SAM and Fe^II^/2OG enzymes involved in streptide (Schramma et al., 2015) and etoposide (Lau and Sattely, 2015) biosynthesis, respectively. In contrast to CylK, these enzymes form bonds between arenes and unactivated carbon centers. Because the target sp^3^ coupling sites of their substrates are inherently unactivated, these alkylating enzymes must stringently discriminate between the alkyl C–H coupling site and its chemically equivalent neighbors. In contrast, the CylK-catalyzed reaction leverages the preinstalled alkyl chloride substituent in order to direct reactivity. Therefore, the cylindrocyclophane biosynthetic logic can be described as dividing the task of determining reaction specificity between CylK and its partner halogenase, CylC. This novel halogenase family likely has strict requirements for substrate positioning (Matthews et al., 2009), although it remains to be seen if CylC is promiscuous. This feature of distributing selectivity and alkylation across two enzymes might rationalize why wild-type CylK can accept multiple non-native substrates, whereas active site specificity for unactivated coupling partners might be more stringent.

CylK performs a challenging chemical reaction without precedent in biology. By elucidating its structure, we have begun to unravel the molecular details underlying the use of alkyl halides as cryptic intermediates in natural product biosynthesis. This enzymatic Friedel–Crafts alkylation might also find application in constructing diverse alkyl–arene C–C bonds outside of natural pathways. With structural information about CylK, notably the identification of active site residues, efforts to further expand its substrate specificity and improve its stability via enzyme engineering can begin in earnest. In addition to its unique reactivity, the large size and solvent accessibility of CylK’s active site suggest that it might be engineered to accept diverse and bulky substrates. Furthermore, our structure and proposed mechanism have increased our fundamental understanding of enzymatic interactions with alkyl halides and may inspire the design of biomimetic chemocatalysts that engage secondary alkyl halides in substitution reactions (Park et al., 2017; Brak and Jacobsen, 2013). Finally, armed with the ability to functionally annotate CylK-like genes, we can prioritize orphan biosynthetic gene clusters and producing organisms in order to discover additional structurally unique natural products.

## Materials and methods

**Key resources table:** 

Reagent type (species) or resource	Designation	Source or reference	Identifiers	Additional information
Gene (*Cylindrospermum licheniforme* ATCC 29412)	CylK	GenBank	ARU81125.1	
Strain, strain background (*Escherichia coli*)	BL21(DE3)	Invitrogen	C6000-03	
Strain, strain background (*E. coli*)	BL21 Gold CodonPlus (DE3) RIL	Agilent	230245	
Peptide, recombinant protein	Strep-Tactin HRP, anti-Strep-HRP (*Streptomyces avidinii*)	IBA Life Sciences	2-1502-001	(1:25,000)
Recombinant DNA reagent	pET-His6-Sumo-CylK-Strep (plasmid)	Schultz et al., 2019		
Recombinant DNA reagent	pPR-IBA1-CylK (plasmid)	Nakamura et al., 2017		
Recombinant DNA reagent	pPR-IBA1-CylK K78E (plasmid)	This work, constructed by GENEWIZ		Replaced AAG with GAG at position 232
Recombinant DNA reagent	pPR-IBA1-CylK D96A (plasmid)	This work, constructed by GENEWIZ		Replaced GAT with GCT at position 286
Recombinant DNA reagent	pPR-IBA1-CylK R105A (plasmid)	This work, constructed by GENEWIZ		Replaced CGT with GCC at position 313
Recombinant DNA reagent	pPR-IBA1-CylK Y473A (plasmid)	This work, constructed by GENEWIZ		Replaced TAT with GCT at position 1,417
Recombinant DNA reagent	pPR-IBA1-CylK S265A (plasmid)	This work, constructed by GENEWIZ		Replaced AGT with GCC at position 793
Recombinant DNA reagent	pPR-IBA1-CylK S266A (plasmid)	This work, constructed by GENEWIZ		Replaced TCC with GCC at position 796
Recombinant DNA reagent	pPR-IBA1-CylK T267A (plasmid)	This work, constructed by GENEWIZ		Replaced ACC with GCT at position 799
Recombinant DNA reagent	pPR-IBA1-CylK S324A (plasmid)	This work, constructed by GENEWIZ		Replaced AGT with GCT at position 970
Recombinant DNA reagent	pPR-IBA1-CylK K377A (plasmid)	This work, constructed by GENEWIZ		Replaced AAA with GCC at position 1129
Recombinant DNA reagent	pPR-IBA1-CylK T379A (plasmid)	This work, constructed by GENEWIZ		Replaced ACC with GCT at position 1135
Recombinant DNA reagent	pPR-IBA1-CylK D502A (plasmid)	This work, constructed by GENEWIZ		Replaced GAC with GCC at position 1504
Recombinant DNA reagent	pPR-IBA1-CylK E504A (plasmid)	This work, constructed by GENEWIZ		Replaced GAA with GCC at position 1510
Recombinant DNA reagent	pPR-IBA1-CylK L558A (plasmid)	This work, constructed by GENEWIZ		Replaced TTA with GCT at position 1672
Recombinant DNA reagent	pPR-IBA1-CylK D559A (plasmid)	This work, constructed by GENEWIZ		Replaced GAT with GCT at position 1675
Recombinant DNA reagent	pPR-IBA1-CylK D562A (plasmid)	This work, constructed by GENEWIZ		Replaced GAT with GCC at position 1684
Recombinant DNA reagent	pPR-IBA1-CylK T616A (plasmid)	This work, constructed by GENEWIZ		Replaced ACG with GCT at position 1846
Recombinant DNA reagent	pPR-IBA1-CylK T618A (plasmid)	This work, constructed by GENEWIZ		Replaced ACT with GCT at position 1852
Recombinant DNA reagent	pPR-IBA1-CylK D440A (plasmid)	This work, constructed by GENEWIZ		Replaced GAC with GCC at position 1318
Recombinant DNA reagent	pPR-IBA1-CylK L438A (plasmid)	This work, constructed by GENEWIZ		Replaced CTT with GCT at position 1312
Recombinant DNA reagent	pPR-IBA1-CylK E374A (plasmid)	This work, constructed by GENEWIZ		Replaced GAA with GCT at position 1120
Recombinant DNA reagent	pPR-IBA1-CylK F499A (plasmid)	This work, constructed by GENEWIZ		Replaced TTT with GCC at position 1495
Recombinant DNA reagent	pPR-IBA1-CylK N334A (plasmid)	This work, constructed by GENEWIZ		Replaced AAC with GCC at position 1000
Recombinant DNA reagent	pPR-IBA1-CylK S318A (plasmid)	This work, constructed by GENEWIZ		Replaced AGC with GCC at position 952
Recombinant DNA reagent	pPR-IBA1-CylK R105K (plasmid)	This work, constructed by GENEWIZ		Replaced CGT with AAG at position 313
Recombinant DNA reagent	pPR-IBA1-CylK Y473F (plasmid)	This work, constructed by GENEWIZ		Replaced TAT with TTT at position 1417
Recombinant DNA reagent	pPR-IBA1-CylK D440N (plasmid)	This work, constructed by GENEWIZ		Replaced GAC with AAC at position 1318
Software, algorithm	HKL2000	Otwinowski and Minor, 1997	http://www.hkl-xray.com/hkl-2000; RRID:SCR_015547	
Software, algorithm	Phaser	McCoy et al., 2007	https://www.phenix-online.org/documentation/reference/phaser.html; RRID:SCR_014219	
Software, algorithm	Phenix	Liebschner et al., 2019	https://www.phenix-online.org/; RRID:SCR_014224	
Software, algorithm	Coot	Emsley and Cowtan, 2004	http://www2.mrc-lmb.cam.ac.uk/personal/pemsley/coot/; RRID:SCR_014222	
Software, algorithm	CCP4 (FFT, CAD)	Winn et al., 2011	http://www.ccp4.ac.uk/; RRID:SCR_007255	
Software, algorithm	Refmac5	Vagin et al., 2004	http://www.ccp4.ac.uk/html/refmac5/description.html; RRID:SCR_014225	
Software, algorithm	PyMOL	The PyMOL Molecular Graphics System, version 2.0 Schrödinger, LLC	http://www.pymol.org/; RRID:SCR_000305	

### Protein expression and purification

CylK was expressed and purified for crystallographic characterization according to a modified literature method (Schultz et al., 2019). Following overexpression in *Escherichia coli* BL21(DE3) as a SUMO fusion construct, cell pellets were resuspended in lysis buffer (20 mM Tris pH 8.0, 500 mM NaCl, 10 mM MgCl_2_, 10 mM CaCl_2_) supplemented with an EDTA-free Pierce Protease Inhibitor Tablet. Cells were lysed using a continuous-flow homogenizer (Avestin EmulsiFlex-C3). The pellet was collected by centrifugation (20,000 × *g* for 30 min) and resuspended in lysis buffer supplemented with 6 M urea and 5 mM imidazole. The denatured enzyme solution was clarified twice by centrifugation (20,000 × *g* for 15 min), and the supernatant was applied to 20 mL of Ni NTA resin. The column was washed with lysis buffer containing 6 M urea and 5 mM imidazole. Bound species were eluted with lysis buffer containing 6 M urea and 250 mM imidazole. All subsequent steps were carried out at 4°C. The enzyme was refolded by sequentially dialyzing this solution against buffer 1 (lysis buffer supplemented with 3 M urea) overnight, buffer 2 (lysis buffer lacking urea) for 4 hr, and buffer 3 (lysis buffer supplemented with 5% glycerol) for 4 hr. Following dialysis, the refolded enzyme solution was centrifuged (100,000 × *g* for 30 min), and the supernatant was collected and incubated overnight with ULP Protease (1:25 w/w). Then, following a subtractive Ni NTA step, the enzyme was applied to a Superdex 200 30/100 gel filtration column (GE Healthcare) equilibrated in protein storage buffer (20 mM HEPES pH 7.8, 50 mM NaCl, 10 mM MgCl_2_, 10 mM CaCl_2_, 10% glycerol). The fractions containing pure CylK were pooled, concentrated (500–700 µM, determined by extinction coefficient and absorbance at 280 nm), and flash frozen in liquid nitrogen.

### Crystallization and structure determination

Prior to crystallization trials, purified CylK protein in storage buffer was combined with 5-undecylbenzene-1,3-diol **3**, a substrate analog synthesized as described previously (Schultz et al., 2019) and dissolved in protein storage buffer containing 22% DMSO. The final protein solution contained 10 mg/mL CylK and 1.66 mM of **3**. Crystals were obtained by using the hanging drop vapor diffusion method in 2 μL drops mixed in 1:1 ratio with a precipitant solution of 1.8 M sodium malonate, pH 7.0. To aid in obtaining phase information, the native Ca(II) sites in CylK crystals were substituted with a lanthanide via soak in a solution of 2.0 M sodium malonate, 100 mM terbium(III) chloride for 8 min at room temperature. The Tb-soaked crystals were transferred to a cryoprotectant solution containing 2.0 M sodium malonate supplemented with 20% ethylene glycol, mounted on rayon loops, and flash frozen by direct plunge into liquid nitrogen. Diffraction datasets were collected at 1.6314 Å (Tb anomalous) and 0. 97872 Å (native) on these crystals at beamlines 23-ID-B (General Medical Sciences and Cancer Institutes Collaborative Access Team [GM/CA-CAT], Advanced Photon Source [APS]) and 21-ID-F (Life Sciences Collaborative Access Team [LS-CAT], APS), respectively. Diffraction datasets were processed with the HKL2000 software package (Otwinowski and Minor, 1997). The structure was solved by using the MR-SAD method. PHASER-EP (McCoy et al., 2007; Read and McCoy, 2011), as implemented within the PHENIX software package (Liebschner et al., 2019), was used to calculate initial phases. A polyalanine model generated from the core β-propeller domain of *Staphylococcus cohnii* streptogramin B lyase (PDB accession code 2QC5, with all loops and secondary structure connections truncated) (Lipka et al., 2008) was used as the initial search model for MR. PHENIX.autobuild generated an initial model containing 504 residues in 15 chain fragments with initial R_work_/R_free_ values of 29.9%/37.4%. The model was iteratively improved via refinement in Refmac5 against a native dataset and model building in Coot (Emsley and Cowtan, 2004), yielding R_work_/R_free_ values of 16.4%/19.8% (Table 1). The final model contains residues 7–45, 49–392, and 413–662 with 11 Ca^2+^ ions, 2 Mg^2+^ ions, 1 Cl^-^ ion, and 339 water molecules. Tb ions were not modeled because they were not present at high occupancy. Ramachandran analysis (Liebschner et al., 2019) indicates a single outlier, Val620. Although **3** was required for CylK crystallization, and weak electron density resembling the compound could be found at several points near the surface of the protein, likely mediating crystal lattice contacts, we could not confidently model the alkyl resorcinol. No density for **3** could be identified in cavity 1, the putative active site of CylK.

To identify possible alkyl chloride-binding sites within the protein, CylK crystals were soaked in 2 M sodium malonate with 500 mM NaBr for up to 1 min. The Br-soaked crystals were transferred to a cryoprotectant solution consisting of the soak solution supplemented with 20% ethylene glycol. Diffraction datasets were collected at 0.9184 Å (Br anomalous) and 0.97872 Å (native) on these crystals at beamlines 23-ID-B (GM/CA-CAT, APS) and 21-ID-F (LS-CAT, APS), respectively. Phase information was obtained by MR using the CylK model as the initial search model in Phaser MR, implemented within CCP4. The model was iteratively built and refined in Coot and Refmac5, respectively, resulting in a R_work_/R_free_ of 16.9%/19.8% (Table 5). The final model contains residues 7–393 and 410–667 with 10 Ca^2+^ ions, 2 Mg^2+^ ions, 1 Na^+^ ion, 4 Br^–^ ions, and 403 water molecules. Anomalous maps were generated using CAD and FFT programs implemented within CCP4. Figures were generated in PyMOL (Schrödinger, LLC). The information in Tables 1 and 5 was generated using HKL2000 (data collection statistics), Refmac5 (refinement statistics including resolution limits, R_work_/R_free_, rms deviations), the PBD validation server (no. of atoms), and PHENIX (*B*-factors, Molprobity clashscore, rotamer outliers, Ramachandran statistics) software packages.

### Mutant enzyme activity screening

CylK mutants were expressed and assayed for activity according to a modified literature method (Schultz et al., 2019). Point mutants in plasmid pPR-IBA1-CylK were synthesized and sequence verified by GENEWIZ, South Plainfield, NJ. Enzyme activity assays using substrate pair **3 + 4** were carried out as follows. Chloro-SNAc electrophile **4** was accessed as described previously (Schultz et al., 2019). Respective mutant, wild-type, and negative control plasmids (Addgene #31122) were freshly transformed into electrocompetent *E. coli* BL21 Gold CodonPlus (DE3) RIL cells (Agilent), and an individual colony was used to directly inoculate 1 mL cultures of LB medium supplemented with 100 μg/mL ampicillin or carbenicillin and 34 μg/mL chloramphenicol in a 96 deep well plate (VWR); sterility was maintained with standard techniques and a gas-permeable rayon film (VWR). The liquid culture plate was incubated for 5 hr at 37°C and 250 rpm shaking. After 5 hr, and confirming visible growth in each well, the liquid culture plate was cooled to 15°C, maintaining shaking. Following an additional 30–45 min of incubation, protein expression was induced with 250 μM IPTG. The cultures were incubated for 4 hr at 15°C with 250 rpm shaking. Following expression, 10 μL of each culture was removed from each well and subcultured for liquid culture RCA-based DNA sequencing to confirm identity and rule out culture cross-contamination. The remaining cultures were concentrated ~10× by centrifugation (3220 × *g* for 10 min) and resuspended in ~200 μL of spent media supernatant. Reactions were initiated in opaque 96-well plates (Costar) by combining 100 μL of concentrated cell suspension with 2 μL of lysozyme mix (9 mg lysozyme and 1 mg EDTA-free Pierce Protease Inhibitor Tablet per 500 μL of 500 mM Tris, 50 mM EDTA, pH 8.0) and monoalkylation substrates in DMSO for a final concentration of nucleophile **3** at 150 μM, electrophile **4** at 300 μM, and DMSO at 3.4%. Reaction plates were sealed with aluminum seals (VWR) and incubated at 37°C with 190 rpm shaking for 20 hr. Reactions were quenched with the addition of 1:1 methanol/acetonitrile (200 μL) and centrifuged (3220 × *g* for 15 min). The supernatant was then subjected to liquid chromatography-mass spectrometry (LC-MS) to measure ion abundance of product **5** (C_31_H_53_ClNO_4_S [M + H^+^] = 536.3768 ± 5 ppm) and starting material **4** (C_14_H_26_ClNO_2_S [M + H^+^] = 308.1446 ± 5 ppm). LC-MS conditions were as published previously (Schultz et al., 2019). Reactions were repeated in biological triplicate.

Enzyme activity on native substrates **1** or **2** was assayed as follows. Substrate **1** and intermediate **2** were accessed chemoenzymatically as described previously (Nakamura et al., 2017). Care was taken to minimize glycerol in substrate stocks because it was determined to inhibit CylK activity. Respective mutant and wild-type plasmids were freshly transformed into electrocompetent *E. coli* BL21 Gold CodonPlus (DE3) RIL cells (Agilent), and 5–10 colonies were used to directly inoculate 100 mL cultures in LB medium supplemented with 100 μg/mL ampicillin or carbenicillin and 34 μg/mL chloramphenicol. The liquid cultures were incubated at 37°C and 190 rpm shaking. At OD400 = 0.4, the cultures were cooled to 15°C, maintaining shaking. Following an additional 1 hr, protein expression was induced with 250 μM IPTG. The cultures were then incubated for 4 hr at 15°C with 190 rpm shaking. Following expression, cell pellets were collected by centrifugation (3220 × *g* for 10 min) and resuspended in ~2 mL of assay buffer (20 mM HEPES pH 7.8, 100 mM NaCl, 10 mM MgCl_2_, 5 mM CaCl_2_). Cell concentration was normalized across mutants by measuring OD600 and diluting with an appropriate amount of assay buffer. A 600 μL aliquot of each mutant was lysed by sonication (Branson, 25% amplitude, 1 min) on ice. The soluble fraction was collected by centrifugation (16,100 × *g* for 20 min) and analyzed by anti-Strep-HRP (IBA) Western blotting to confirm mutant solubility and approximate protein concentration (Figure 4—figure supplement 2). Reactions were initiated by combining 4.3 μL of **1** or **2** with 25.7 μL of soluble lysate for a final concentration of **1** at 400 μM and 4% DMSO, and **2** at 200 μM and 2% DMSO. Reactions were sealed and incubated at 37°C for 1 or 20 hr. Reactions were quenched with the addition of 1:1 methanol/acetonitrile (60 μL) and centrifuged (6000 × *g* for 15 min). The supernatant was then subjected to analytical high-performance liquid chromatography (HPLC) to determine percent conversion as described previously. Reactions were repeated in biological duplicate, and then repeated again on a second day to minimize any potential variation. Results were consistent on both days.

### Molecular dynamics simulations

Coordinates for the native substrates **1** and **2** were prepared in ACEDRG (Long et al., 2017) from MOL file descriptions of the molecules generated in ChemDraw. Two substrate molecules were docked manually into cavity 1 of CylK using Br1 to place the alkyl chloride component and interactions with essential amino acids to guide placement of the second substrate. The resulting CylK complexes were prepared for molecular dynamics (MD) simulations using the Xleap module of AmberTools (Case, 2020). Substrates were parameterized using Antechamber (Wang et al., 2001; Wang et al., 2004) in AmberTools, while the FF14SB forcefield (Maier et al., 2015) was used for protein atoms. Complexes were solvated in a 10 Å octahedral box of TIP3P water (Jorgensen et al., 1983). Sodium and chloride ions were added to neutralize the complexes and achieve a final concentration of 150 mM NaCl. All minimizations and production runs were performed with Amber20 (Case, 2020). Minimization was performed using 5000 steps of steepest descent and 5000 steps of conjugate gradient. In the first round, 500 kcal/mol·Å^2^ restraints on all protein, ligand atoms, as well as the metal ion cofactors. Restraints were reduced to 100 kcal/mol·Å^2^ for an additional round of solvent minimization. Next, restraints retained only ligands and metal ions for a third round of minimization. Finally, all restraints were removed for the last stage of minimization. After minimization, a 100 ps run was used to heat complexes from 0 to 300 K using constant volume periodic boundaries with 10 kcal/mol·Å^2^ restraints on all solute atoms. Equilibration was performed using two 10 ns runs (10 kcal/mol·Å^2^ and 1 kcal/mol·Å^2^ restraints) on protein, ligand, and metal ions. Following equilibration, simulations were run as either restrained or unrestrained. For restrained simulations, we obtained 100 ns trajectories with 1 kcal/mol·Å^2^ restraints on protein atoms. Unrestrained simulations (100 ns) were run with all restraints removed.

### Bioinformatic analysis

The CylK (GenBank: ARU81125.1) and BrtB (GenBank: AOH72618.1) protein sequences were independently used in a Basic Local Alignment Search Tool (BLAST) search of the Joint Genome Institute-Integrated Microbial Genomes & Microbiomes (IMG-JGI; Chen et al., 2021) database of all isolates, metagenome-assembled genomes (MAGs), and single-amplified genomes (SAGs). Each BLAST search resulted in 500 hits (>24% amino acid sequence identity) and were merged to form a nonredundant list of 715 enzyme candidates (Supplementary file 1). Parent enzyme sequences (CylK, BrtB) and likely CylK homologs (MerH, CabK, CylK from *Cylindrospermum stagnale*) were added to this dataset. A targeted BLAST search of the genomes from this list was performed to identify all putative halogenases (CylC-like, Fe^II^/2OG, SAM-dependent, flavin-dependent, and haloperoxidase) and resorcinol-forming enzymes (CylI, BrtC, BrtD). All non-CylC halogenases were confirmed to contain key catalytic residues and examined for co-localization with CylK. All candidate CylC halogenases were included, although the overwhelming majority (63 of 78 enzymes) were fewer than 18 genes away from CylK (Supplementary file 2). CylC’s catalytic residues remain unknown. MAR-forming CylI homologs were included if clustered with CylK; 11 of 54 candidate enzymes were less than 12 genes away from CylK (Supplementary file 2). Next, candidate CylK enzymes were filtered by constructing an individual MUSCLE alignment (Edgar, 2004) of each hit to the CylK parent sequence and identifying at least 135 amino acid residues before the C-terminal domain. Lys240 or its aligned equivalent was defined as the junction point between protein domains. This analysis resulted in 286 unique protein sequences (Supplementary file 2) that were aligned (MUSCLE, EMBL-EBI), sites containing gaps at ≥90% of sequences were removed, and a maximum-likelihood phylogenetic tree was constructed using IQ-TREE v1.6.12 (Nguyen et al., 2015) and visualized with FigTree v1.4.4. The best-fit model (VT + F + G4) was selected by ModelFinder (Kalyaanamoorthy et al., 2017). Bootstrap values were calculated using the UFBoot2 method (Hoang et al., 2018). These candidate enzymes were analyzed for the presence or absence of Arg105 and Tyr473 residues by constructing an individual MUSCLE alignment of each hit to the CylK parent sequence. The results were manually mapped onto the phylogenetic tree. The subset CylK homologs with both residues were MUSCLE aligned and visualized with ConSurf (Ashkenazy et al., 2016). The code used to perform iterative MUSCLE alignments is available at https://github.com/nbraffman/CylK-homologs, (copy archived at swh:1:rev:732a38ef8ab73988203ea1933158238ea90361b0; Braffman, 2021).

## Data Availability

Diffraction data have been deposited in the PDB under the accession codes 7RON, 7ROO. All other data generated or analyzed during this study and included in the manuscript and supporting files; Source Data files have been provided for Figure 4. The following datasets were generated: RuskoskiTB BoalAK 2022Crystal structure of the Friedel-Crafts alkylating enzyme CylK from Cylindospermum licheniformeRCSB Protein Data Bank7RON RuskoskiTB BoalAK 2022Crystal structure of Friedel-Crafts alkylating enzyme CylK from Cylindospermum licheniforme with bromideRCSB Protein Data Bank7ROO

## Additional files

Supplementary file 1. Catalog of 715 protein sequences with homology to CylK and/or BrtB from the IMG-JGI database.

Supplementary file 2. Annotated table of putative CylK homologs that contain N-terminal fusions or are encoded by organisms with CylC- or CylI-like enzymes.

Transparent reporting form

## References

[bib1] Adak S, Moore BS (2021). Cryptic halogenation reactions in natural product biosynthesis. Natural Product Reports.

[bib2] Ashkenazy H, Abadi S, Martz E, Chay O, Mayrose I, Pupko T, Ben-Tal N (2016). ConSurf 2016: an improved methodology to estimate and visualize evolutionary conservation in macromolecules. Nucleic Acids Research.

[bib3] Braffman NR (2021). GitHub.

[bib4] Brak K, Jacobsen EN (2013). Asymmetric ion-pairing catalysis. Angewandte Chemie (International Ed. in English).

[bib5] Buchinger E, Knudsen DH, Behrens MA, Pedersen JS, Aarstad OA, Tøndervik A, Valla S, Skjåk-Bræk G, Wimmer R, Aachmann FL (2014). Structural and Functional Characterization of the R-modules in Alginate C-5 Epimerases AlgE4 and AlgE6 from Azotobacter vinelandii. Journal of Biological Chemistry.

[bib6] Case H (2020). AMBER 2020.

[bib7] Chen CK-M, Chan N-L, Wang AH-J (2011). The many blades of the β-propeller proteins: conserved but versatile. Trends in Biochemical Sciences.

[bib8] Chen I-MA, Chu K, Palaniappan K, Ratner A, Huang J, Huntemann M, Hajek P, Ritter S, Varghese N, Seshadri R, Roux S, Woyke T, Eloe-Fadrosh EA, Ivanova NN, Kyrpides NC (2021). The IMG/M data management and analysis system v.6.0: new tools and advanced capabilities. Nucleic Acids Research.

[bib9] Dong C, Huang F, Deng H, Schaffrath C, Spencer JB, O’Hagan D, Naismith JH (2004). Crystal structure and mechanism of a bacterial fluorinating enzyme. Nature.

[bib10] Edgar RC (2004). MUSCLE: multiple sequence alignment with high accuracy and high throughput. Nucleic Acids Research.

[bib11] Emsley P, Cowtan K (2004). Coot: model-building tools for molecular graphics. Acta Crystallographica. Section D, Biological Crystallography.

[bib12] Eustáquio AS, Pojer F, Noel JP, Moore BS (2008). Discovery and characterization of a marine bacterial SAM-dependent chlorinase. Nature Chemical Biology.

[bib13] Friedel C, Crafts JM (1877). Sur une nouvelle méthode générale de synthèse d’hydrocarbures, d’acétones, etc. Comptes Rendus Des Séances.

[bib14] Garau C, Quiñonero D, Frontera A, Ballester P, Costa A, Deyà PM (2003). Anion–π interactions: must the aromatic ring be electron deficient?. New Journal of Chemistry.

[bib15] Gáspári Z (2020). Structural Bioinformatics.

[bib16] Ho BK, Gruswitz F (2008). HOLLOW: generating accurate representations of channel and interior surfaces in molecular structures. BMC Structural Biology.

[bib17] Hoang DT, Chernomor O, von Haeseler A, Minh BQ, Vinh LS (2018). UFBoot2: Improving the Ultrafast Bootstrap Approximation. Molecular Biology and Evolution.

[bib18] Jorgensen WL, Chandrasekhar J, Madura JD, Impey RW, Klein ML (1983). Comparison of simple potential functions for simulating liquid water. The Journal of Chemical Physics.

[bib19] Kalyaanamoorthy S, Minh BQ, Wong TKF, von Haeseler A, Jermiin LS (2017). ModelFinder: fast model selection for accurate phylogenetic estimates. Nature Methods.

[bib20] Korczynska M, Mukhtar TA, Wright GD, Berghuis AM (2007). Structural basis for streptogramin B resistance in *Staphylococcus aureus* by virginiamycin B lyase. PNAS.

[bib21] Lau W, Sattely ES (2015). Six enzymes from mayapple that complete the biosynthetic pathway to the etoposide aglycone. Science (New York, N.Y.).

[bib22] Leão PN, Nakamura H, Costa M, Pereira AR, Martins R, Vasconcelos V, Gerwick WH, Balskus EP (2015). Biosynthesis-assisted structural elucidation of the bartolosides, chlorinated aromatic glycolipids from cyanobacteria. Angewandte Chemie (International Ed. in English).

[bib23] Liebschner D, Afonine PV, Baker ML, Bunkóczi G, Chen VB, Croll TI, Hintze B, Hung LW, Jain S, McCoy AJ, Moriarty NW, Oeffner RD, Poon BK, Prisant MG, Read RJ, Richardson JS, Richardson DC, Sammito MD, Sobolev OV, Stockwell DH, Terwilliger TC, Urzhumtsev AG, Videau LL, Williams CJ, Adams PD (2019). Macromolecular structure determination using X-rays, neutrons and electrons: recent developments in Phenix. Acta Crystallographica. Section D, Structural Biology.

[bib24] Linhartová I, Bumba L, Mašín J, Basler M, Osička R, Kamanová J, Procházková K, Adkins I, Hejnová-Holubová J, Sadílková L, Morová J, Šebo P (2010). RTX proteins: a highly diverse family secreted by a common mechanism. FEMS Microbiology Reviews.

[bib25] Lipka M, Filipek R, Bochtler M (2008). Crystal structure and mechanism of the Staphylococcus cohnii virginiamycin B lyase (Vgb). Biochemistry.

[bib26] Long F, Nicholls RA, Emsley P, Graǽulis S, Merkys A, Vaitkus A, Murshudov GN (2017). AceDRG: a stereochemical description generator for ligands. Acta Crystallographica. Section D, Structural Biology.

[bib27] Maier JA, Martinez C, Kasavajhala K, Wickstrom L, Hauser KE, Simmerling C (2015). ff14SB: Improving the Accuracy of Protein Side Chain and Backbone Parameters from ff99SB. Journal of Chemical Theory and Computation.

[bib28] Martins TP, Rouger C, Glasser NR, Freitas S, de Fraissinette NB, Balskus EP, Tasdemir D, Leão PN (2019). Chemistry, bioactivity and biosynthesis of cyanobacterial alkylresorcinols. Natural Product Reports.

[bib29] Matthews ML, Neumann CS, Miles LA, Grove TL, Booker SJ, Krebs C, Walsh CT, Bollinger JM (2009). Substrate positioning controls the partition between halogenation and hydroxylation in the aliphatic halogenase, SyrB2. PNAS.

[bib30] May DS, Chen WL, Lantvit DD, Zhang X, Krunic A, Burdette JE, Eustaquio A, Orjala J (2017). Merocyclophanes C and D from the Cultured Freshwater Cyanobacterium Nostoc sp. (UIC 10110). Journal of Natural Products.

[bib31] McCoy AJ, Grosse-Kunstleve RW, Adams PD, Winn MD, Storoni LC, Read RJ (2007). Phaser crystallographic software. Journal of Applied Crystallography.

[bib32] Meier R, Drepper T, Svensson V, Jaeger KE, Baumann U (2007). A calcium-gated lid and A large beta-roll sandwich are revealed by the crystal structure of extracellular lipase from Serratia marcescens. The Journal of Biological Chemistry.

[bib33] Messing SAJ, Gabelli SB, Echeverria I, Vogel JT, Guan JC, Tan BC, Klee HJ, McCarty DR, Amzel LM (2010). Structural Insights into Maize Viviparous14, a Key Enzyme in the Biosynthesis of the Phytohormone Abscisic Acid. The Plant Cell.

[bib34] Motlova L, Klimova N, Fiser R, Sebo P, Bumba L (2020). Continuous Assembly of β-Roll Structures Is Implicated in the Type I-Dependent Secretion of Large Repeat-in-Toxins (RTX) Proteins. Journal of Molecular Biology.

[bib35] Nakamura H, Schultz EE, Balskus EP (2017). A new strategy for aromatic ring alkylation in cylindrocyclophane biosynthesis. Nature Chemical Biology.

[bib36] Neumann CS, Fujimori DG, Walsh CT (2008). Halogenation strategies in natural product biosynthesis. Chemistry & Biology.

[bib37] Nguyen L-T, Schmidt HA, von Haeseler A, Minh BQ (2015). IQ-TREE: A fast and effective stochastic algorithm for estimating maximum-likelihood phylogenies. Molecular Biology and Evolution.

[bib38] Otwinowski Z, Minor W (1997). Processing of X-ray diffraction data collected in oscillation mode. Methods in Enzymology.

[bib39] Parisini E, Metrangolo P, Pilati T, Resnati G, Terraneo G (2011). Halogen bonding in halocarbon-protein complexes: a structural survey. Chemical Society Reviews.

[bib40] Park Y, Harper KC, Kuhl N, Kwan EE, Liu RY, Jacobsen EN (2017). Macrocyclic bis-thioureas catalyze stereospecific glycosylation reactions. Science (New York, N.Y.).

[bib41] Pavkov‐Keller T, Schmidt NG, Żądło‐Dobrowolska A, Kroutil W, Gruber K (2018). Structure and Catalytic Mechanism of a Bacterial Friedel–Crafts Acylase. ChemBioChem.

[bib42] Pomowski A, Zumft WG, Kroneck PMH, Einsle O (2011). N2O binding at a [4Cu:2S] copper–sulphur cluster in nitrous oxide reductase. Nature.

[bib43] Preisitsch M, Harmrolfs K, Pham HTL, Heiden SE, Füssel A, Wiesner C, Pretsch A, Swiatecka-Hagenbruch M, Niedermeyer THJ, Müller R, Mundt S (2015). Anti-MRSA-acting carbamidocyclophanes H-L from the Vietnamese cyanobacterium Nostoc sp. CAVN2. The Journal of Antibiotics.

[bib44] Preisitsch M, Niedermeyer THJ, Heiden SE, Neidhardt I, Kumpfmüller J, Wurster M, Harmrolfs K, Wiesner C, Enke H, Müller R, Mundt S (2016). Cylindrofridins A-C, Linear Cylindrocyclophane-Related Alkylresorcinols from the Cyanobacterium Cylindrospermum stagnale. Journal of Natural Products.

[bib45] Ravaud S, Gouet P, Haser R, Aghajari N (2003). Probing the role of divalent metal ions in a bacterial psychrophilic metalloprotease: binding studies of an enzyme in the crystalline state by X-ray crystallography. Journal of Bacteriology.

[bib46] Read RJ, McCoy AJ (2011). Using SAD data in Phaser. Acta Crystallographica. Section D, Biological Crystallography.

[bib47] Reis JPA, Figueiredo SAC, Sousa ML, Leão PN (2020). BrtB is an O-alkylating enzyme that generates fatty acid-bartoloside esters. Nature Communications.

[bib48] Roberts RM, Khalaf AA (1984). Friedel–Crafts Alkylation Chemistry: A Century of Discovery.

[bib49] Rudolph A, Lautens M (2009). Secondary alkyl halides in transition-metal-catalyzed cross-coupling reactions. Angewandte Chemie (International Ed. in English).

[bib50] Schapira M, Tyers M, Torrent M, Arrowsmith CH (2017). WD40 repeat domain proteins: a novel target class?. Nature Reviews. Drug Discovery.

[bib51] Schottel BL, Chifotides HT, Dunbar KR (2008). Anion-π interactions. Chem. Soc. Rev.

[bib52] Schramma KR, Bushin LB, Seyedsayamdost MR (2015). Structure and biosynthesis of a macrocyclic peptide containing an unprecedented lysine-to-tryptophan crosslink. Nature Chemistry.

[bib53] Schultz EE, Braffman NR, Luescher MU, Hager HH, Balskus EP (2019). Biocatalytic Friedel-Crafts Alkylation Using a Promiscuous Biosynthetic Enzyme. Angewandte Chemie (International Ed. in English).

[bib54] Tanaka S, Saito K, Chon H, Matsumura H, Koga Y, Takano K, Kanaya S (2007). Crystal Structure of Unautoprocessed Precursor of Subtilisin from a Hyperthermophilic Archaeon. Journal of Biological Chemistry.

[bib55] Vagin AA, Steiner RA, Lebedev AA, Potterton L, McNicholas S, Long F, Murshudov GN (2004). REFMAC5 dictionary: organization of prior chemical knowledge and guidelines for its use. Acta Crystallographica. Section D, Biological Crystallography.

[bib56] Vaillancourt FH, Yeh E, Vosburg DA, O’Connor SE, Walsh CT (2005). Cryptic chlorination by a non-haem iron enzyme during cyclopropyl amino acid biosynthesis. Nature.

[bib57] Verschueren KHG, Seljée F, Rozeboom HJ, Kalk KH, Dijkstra BW (1993). Crystallographic analysis of the catalytic mechanism of haloalkane dehalogenase. Nature.

[bib58] von Kügelgen A, Tang H, Hardy GG, Kureisaite-Ciziene D, Brun YV, Stansfeld PJ, Robinson CV, Bharat TAM (2020). In Situ Structure of an Intact Lipopolysaccharide-Bound Bacterial Surface Layer. Cell.

[bib59] Wang J, Wang W, Kollman PA, Case DA (2001). Antechamber: an accessory software package for molecular mechanical calculations. Journal of the American Chemical Society.

[bib60] Wang J, Wolf RM, Caldwell JW, Kollman PA, Case DA (2004). Development and testing of a general amber force field. Journal of Computational Chemistry.

[bib61] Wang HQ, Mou SB, Xiao W, Zhou H, Hou XD, Wang SJ, Wang Q, Gao J, Wei Z, Liu L, Xiang Z (2022). Structural Basis for the Friedel–Crafts Alkylation in Cylindrocyclophane Biosynthesis. ACS Catalysis.

[bib62] Winn MD, Ballard CC, Cowtan KD, Dodson EJ, Emsley P, Evans PR, Keegan RM, Krissinel EB, Leslie AGW, McCoy A, McNicholas SJ, Murshudov GN, Pannu NS, Potterton EA, Powell HR, Read RJ, Vagin A, Wilson KS (2011). Overview of the CCP4 suite and current developments. Acta Crystallographica. Section D, Biological Crystallography.

