# Supplementary figures and images for: Structural basis for an unprecedented enzymatic alkylation in cylindrocyclophane biosynthesis
Source: eLife. 2022 Feb 25;11:e75761. doi: 10.7554/eLife.75761 (PMC8916777; doi:10.7554/eLife.75761)

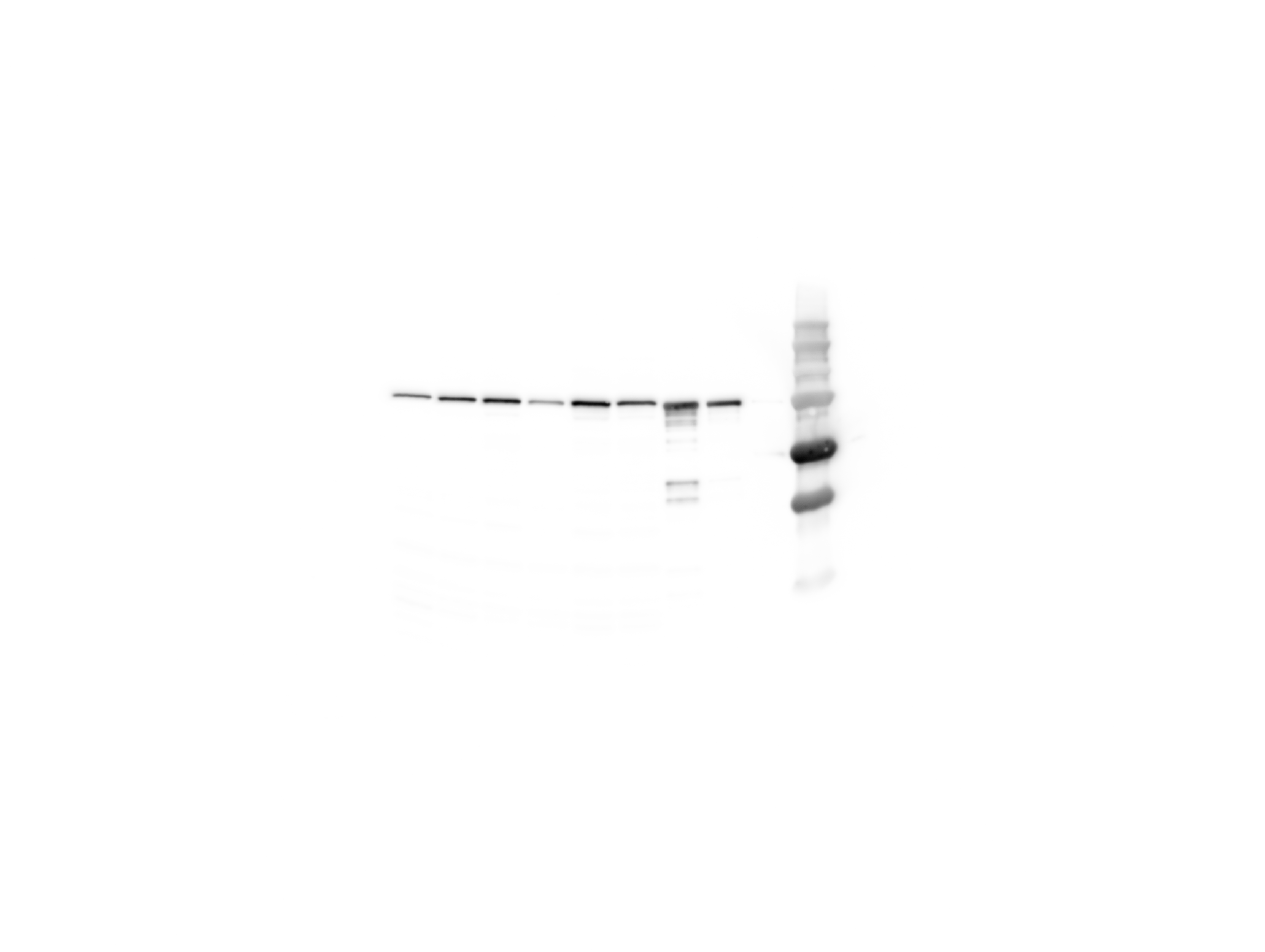

Supplement: Figure 4—source data 1. [file elife-75761-fig4-data1.zip › Figure 4-Source Data 1/Figure 4-Source Data 1.tif]

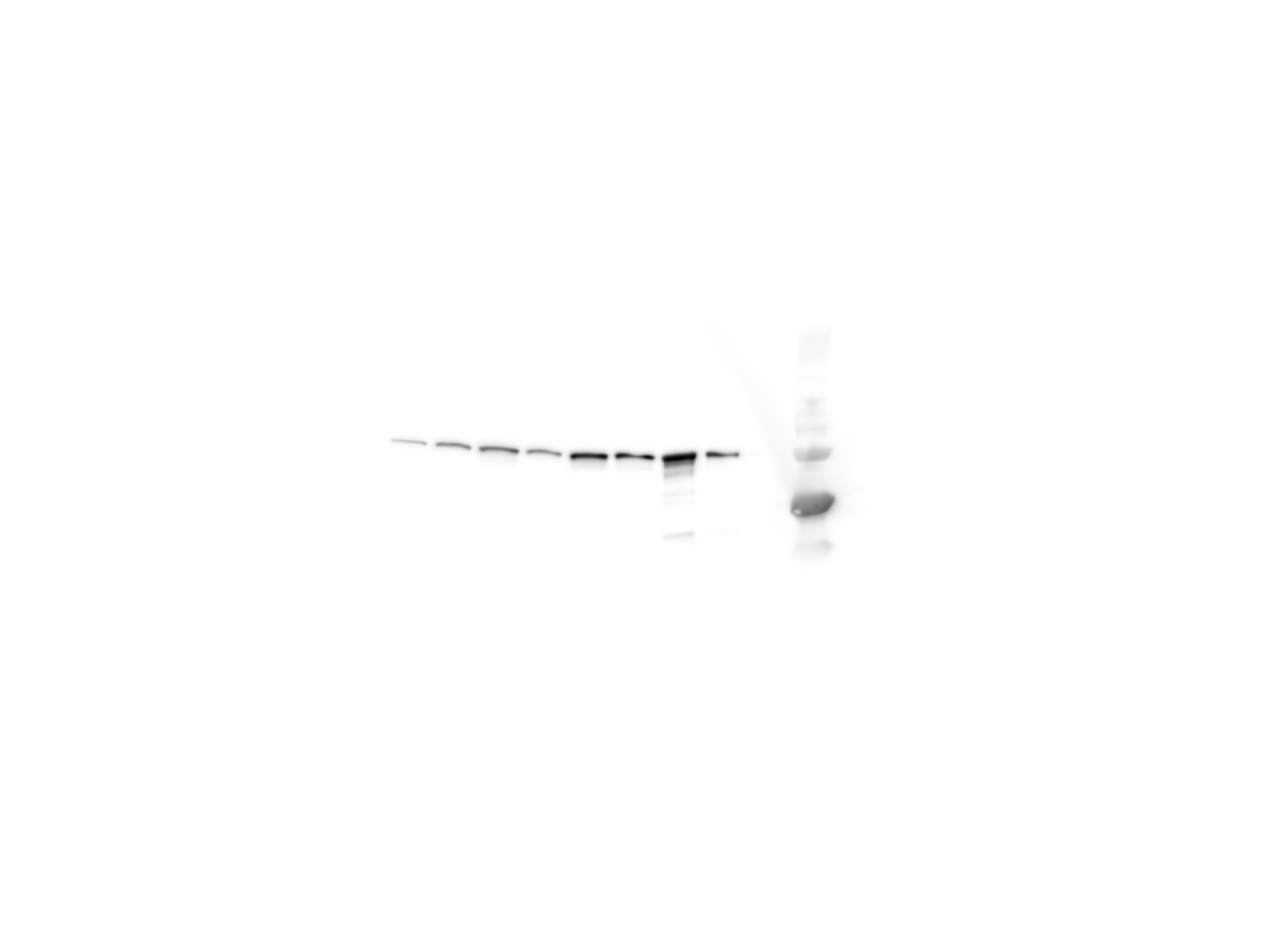

Supplement: Figure 4—source data 2. [file elife-75761-fig4-data2.zip › Figure 4-Source Data 2/Figure 4-Source Data 2.tif]
